# Lyophilized Small Extracellular Vesicles (sEVs) Derived from Human Adipose Stem Cells Maintain Efficacy to Promote Healing in Neuronal Injuries

**DOI:** 10.3390/biomedicines13020275

**Published:** 2025-01-23

**Authors:** Brianna Jones, Rekha Patel, Bangmei Wang, Theresa Evans-Nguyen, Niketa A. Patel

**Affiliations:** 1 Research Service, J.A. Haley Veterans Hospital, Tampa, FL 33612, USA; brianna.jones2@va.gov (B.J.); niketa.patel@va.gov (N.A.P.); rekha.patel1@va.gov (R.P.); bangmei.wang@va.gov (B.W.); 2 Department of Chemistry, College of Arts and Sciences, University of South Florida, Tampa, FL 33612, USAevansnguyen@usf.edu (T.E.-N.); 3 Department of Molecular Medicine, Morsani College of Medicine, University of South Florida, Tampa, FL 33612, USA

**Keywords:** sEVs, exosomes, adipose stem cells, traumatic brain injury, TBI, hASC sEVs, lyophilization, NTA

## Abstract

**Background:** Traumatic brain injury (TBI) occurs in individuals of all ages, predominantly during sports, accidents, and in active military service members. Chronic consequences of TBI include declined cognitive and motor function, dementia, and emotional distress. Small extracellular vesicles (sEVs), previously referred to as exosomes, are nano-sized lipid vesicles that play a role in intercellular communication. Our prior research established the efficacy of sEVs derived from human adipose stem cells (hASC sEVs) in accelerating the healing of brain injuries. The hASC sEVs are a biologic therapeutic and need to be stored at −20 °C or −80 °C. This limits their use in translating to everyday use in clinics or their inclusion in first-aid kits for application immediately after injury. To address this, here we demonstrate that hASC sEVs can be stored at room temperature (RT) for two months post lyophilization. **Methods:** A transmission electron microscope (TEM) and nanoparticle tracking analysis (NTA) were used to validate the morphology of lyophilized RT sEVs. Using in vitro models of neuronal injury mimicking physical injury, inflammation, and oxidative stress, we demonstrate that lyophilized RT hASC sEVs are viable and promote the healing of neuronal injuries. **Results:** The lyophilized sEVs maintain their purity, size, and morphology upon rehydration. Lyophilized, RT stored sEVs showed better efficacy after two months compared with −80 °C stored sEVs. **Conclusions:** RT storage of lyophilized hASC sEVs maintains their efficacy to accelerate the healing of injuries in neuronal cells. This will advance the use of hASC sEVs, bringing them closer to use in clinics, home first-aid kits, and on battlefields by active service members.

## 1. Introduction

Traumatic brain injury (TBI) has exponentially increased in the last few years and affects nearly 50 million people globally. TBI is defined as a disruption or injury to brain function caused by an outside force, such as a blow or jolt to the head or an object entering the brain [1]. The effects from TBI can be either short- or long-term; the long-term effects are more severe and, in some cases, result in early death. Secondary injury, often tied to neuroinflammation, results from the primary brain injury and occurs over time, and it includes chemical, cellular, tissue, and blood vessel changes in the brain. Secondary injuries typically include ischemia, cerebral edema, hypoxia, and more [2]. These injuries can cause secondary cell death, resulting in a decrease in cell proliferation [3,4]. The development of treatments for these injuries is often difficult due to the complexity of progression. These complications stem from pathophysiological and prolonged development of secondary injuries. The most common treatments for secondary injury generally target neuroinflammation; however, there is a still a need for more effective treatments [5]. The impact of TBI may last for several years, and it can leave some individuals disabled or in need of rehospitalization even 10 years after the injury. Thus, the availability of robust treatment options in the hours following TBI is imperative [6,7].

One of the most widely affected demographics of TBI are active military service members and veterans. TBI is known to be the most impactful cause of disabilities related to combat [8]. TBI is a signature injury of the Global War on Terrorism (GWOT) conflicts, including post-9/11 conflicts, Operation Iraqi Freedom (OIF), Operation Enduring Freedom (OEF), and Operation New Dawn (OND) [9,10].

Recently, the definition and nomenclature of extracellular vesicles (EVs) have been updated according to the Information for Studies of Extracellular Vesicles (ISEV) [11]. Small extracellular vesicles (sEVs) are characterized as EVs that are lipid-membrane-bound particles less than 200 nm and released by cells. Exosomes are often identified through surface markers such as the tetraspanins CD9, CD63, CD81. However, research is still ongoing on whether these tetraspanins solely mark exosomes and no other intracellular multivesicular bodies.

Mesenchymal stem cells (MSCs) derived from adipose tissue have become a promising therapeutic for TBI due to their anti-inflammatory and regenerative properties [12]. However, since stem cells can differentiate into different phenotypes based on the stimuli, the secretome was evaluated. We have previously demonstrated that the secretome from human adipose stem cells (hASCs) contains large and small extracellular vesicles (EVs). We further characterized the sEV using the tetraspanin markers CD9, CD63, and CD81, which demonstrated that hASC sEVs (previously referred to as exosomes in our publications), contained long, noncoding RNA that was pivotal to driving the regeneration and repair post brain injury [4]. In this study, we used the hASCs from the same master hASC bank and collected its conditioned media to purify the sEVs and evaluate their efficacy upon lyophilization. Over the past decade, there has been an increased interest in the use of sEVs due to their important role in intercellular communication and drug-delivery applications. The sEVs carry cargo such as proteins, RNA, and DNA that can be transported from cell to cell and serve as biomarkers for disease progression and diagnosis [13]. The sEVs have also become a useful tool as a drug-delivery vehicle. Due to their low toxicity, biocompatibility, and ability to carry small molecules, these vesicles have also proven to be effective in delivering drugs [14,15].

There are various ways to isolate sEVs. The method of isolation can influence the purity, functionality, and performance of sEVs [16,17]. Some of the most common methods used include size exclusion-based methods and ultracentrifugation. Methods that include ultracentrifugation can harm the morphology of the sEVs due to the force and are often tedious. Size exclusion methods can remove larger EVs and contaminating particles that are not vesicles while keeping the sEVs intact [18]. More recently, size exclusion methods have been coupled with other methods such as ultrafiltration to facilitate more efficient removal of bigger particles. In this study, we investigated the efficiency of ExoSpin columns compared with qEV columns for sEV isolation.

**Figure 1:**
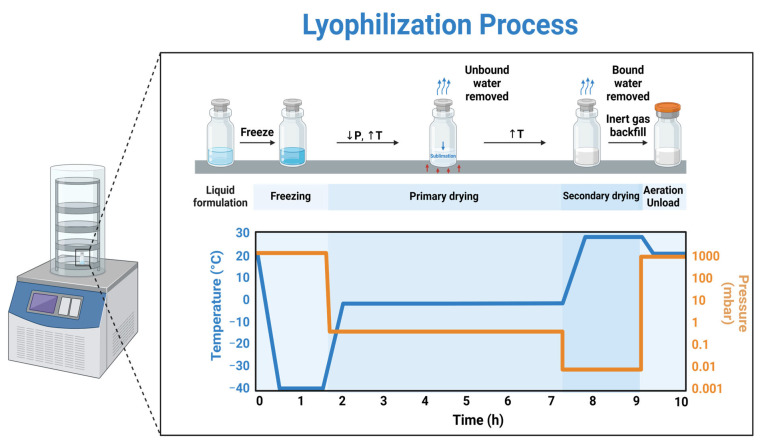
Schematic of the three processes that occur during lyophilization. The publication license can be found in Supplementary File S2 (Created in BioRender. Jones, B. (2024) https://BioRender.com/s06h883; accessed on 15 December 2024).

The hASC sEVs have tremendous potential in treating TBI; however, there remains an issue with storage that limits their use in the clinic and battlefield. The sEVs stored at 4 °C show 100% efficacy up to 1 week. sEVs are usually stored long-term at −80 °C to preserve their size and cargo; however, several studies have reported that storage of EVs and sEVs at −80 °C can cause harm to their morphology and cargo over time, interfering with their performance [19,20]. In addition, rural clinics and battlefield clinics often do not have access to −80 °C ultra-low freezers. To address this issue, lyophilization has been proposed as a storage method at room temperature [21]. Lyophilization (freeze-drying) is a method that removes water from a sample using sublimation and desorption (Figure 1). There are three phases to the lyophilization process. The first phase is freezing, which involves cooling the material below its triple point. This can be achieved by rapidly freezing the sample in a −80 °C freezer for biological samples. The next phase is primary drying or sublimation. During sublimation, the pressure is lowered (↓) and the sample is heated (↑) to −20 °C so the water will sublimate and solidify while under vacuum [22]. The last phase is secondary drying or adsorption. During this phase, water-bound molecules are removed from the material by raising the temperature. During freezing and drying, the bilayer and cargo of the sEVs can be damaged due to the stresses they undergo during these processes. To combat this issue, cryoprotectants may be used [23].

In this study, we used trehalose, a nonreducing disaccharide found in many organisms, to protect the hASC sEVs during lyophilization [24]. We investigated the sEVs’ morphology, size, and concentration as well as protein concentrations post lyophilization and storage at room temperature. We then investigated the in vitro efficacy of the lyophilized hASC sEVs in models of neuronal injury mimicking TBI.

The aim of the study was to (1) compare two widely used EV isolation methods to establish an effective and reproducible isolation method for the extraction of sEVs from hASCs; (2) demonstrate that lyophilization with the addition of trehalose does not change the morphology or size of the sEVs; and (3) compare the efficacy of lyophilized RT stored sEVs with −80 °C stored sEVs using in vitro neuronal injury models.

## 2. Materials and Methods

### 2.1. Cell Culture

Studies were carried out using mouse hippocampal neuronal cell line (HT22) Dr. D.R. Schubert (Salk Institute, La Jolla, CA, USA). HT22 cells were cultured in DMEM (GibcoLifeTechnologies, Waltham, MA, USA: # 11,965–092), 10% FBS (Sigma, St. Louis, MO, USA; #F4135), and 1 × penicillin–streptomycin (Sigma #P4333) at 37 °C and 5% CO_2_. The hASCs (ZenBio, Durham, NC, USA: #ASC-F-SL) were pooled from lean female subcutaneous depo with an average BMI of 27.9. All donors were non-smokers and non-diabetic. For collection of conditioned media (CM) from human adipose stem cells (hASCs), hASCs were grown to 90% confluence (8 × 10^6^ hASCs) in T75 flasks, and then, cell medium was replaced with serum-free mesenchymal stem cell basal media with StemFlex medium kit (GibcoLifeTechnologies, Waltham, MA, USA, # A33494-01). The CM derived from hASCs was collected after 48 h.

### 2.2. sEV Isolation and Size Characterization

The CM (as described in Section 2.1) was then used to isolate sEVs. CM was centrifuged at 3000× *g* for 15 min to remove any hASC cell debris. The CM was then processed to isolate sEVs using 2 methods.

For hASC sEVs isolated using ExoSpin columns, ExoSpin™ (Cell Guidance systems, St. Louis, MO, USA: Catalog EX05) reagent was added to the CM and incubated for 20 min at room temperature. The mixture was centrifuged at 1500× *g* for 30 min to remove large EVs by precipitation, and the supernatant was added to the top of the ExoSpin column and centrifuged at 50× *g* for 60 s. The sEVs were eluted in PBS by centrifugation.

For sEVs isolated using qEV columns (35 nm from IZON, Bellaire, TX, USA), CM was concentrated using a 10 kDa molecular weight cut-off filter (MWCO) which retains the large EVS and allows only EVs smaller than 10 kDa to pass through the pores. qEV columns were prepared following the manufacturer’s instructions using PBS as a buffer. The qEV column was mounted on the Automated Fraction Collector (AFC, from IZON, Bellaire, TX, USA), which enabled reproducible and exact collection of fractions by size exclusion chromatography. The concentrated CM was then added to the prepared qEV columns. The sEVs were eluted in PBS, and fractions were collected every 500 μL until a pink color was seen eluting from the column (end of sample collection). Nanoparticle tracking analysis (NTA3.1, Build 3.1.46 RRID SCR-014239) was used to analyze size and concentration of sEVs in each fraction. The fractions containing a single peak of sEV particles within 35–200 nm size were identified and used in the TEM to image the morphology and in the experimental setups.

### 2.3. Transmission Electron Microscopy (TEM)

For the hASC sEVs, 4 μL at a concentration of 1 × 10^8^ particles/mL was placed on a carbon-filled coated copper mesh grid. The sample was then incubated for 10 min at room temperature. The excess liquid was then removed from the grid using filter paper. One drop of 0.2 micron filtered, boiled distilled water was placed on the grid and removed three times to rinse off PBS. Sample was left to dry overnight. Samples were then imaged using a JEOL 1400 transmission electron microscope at 100 k× magnification. All TEM images are the original images. To select which TEM images would be used, the following criteria were followed: (1) The low-magnification image should show two or more sEVs in the field or include minimal debris from the sEV; (2) the size of the particles present should be between 30 and 150 nm and have a spherical or cup shape with a distinct, well-defined border; (3) the image should have good contrast to show the membrane of the vesicle; and (4) the particles present should have electron-dense areas inside of the defined border to indicate the cargo is present [25].

### 2.4. Lyophilization

A total of 500 μL of isolated sEVs at a concentration of 4.5 × 10^8^ particles/mL was buffer exchanged to 50 mM trehalose in water using 50 kDa MWCO filters. The buffer-exchanged samples were aliquoted into ten 50 μL samples, and then rapidly frozen. Samples were lyophilized overnight using a FreeZone 4.5 liter Console Freeze Dry System from Labconco Corporation, Kansas City, MO, USA and stored at room temperature for 2–8 weeks. Before use, lyophilized samples were rehydrated with PBS to their original volume and used in the experiments. For lyophilized sEVs without trehalose, sEV samples were buffer-exchanged to water and rapidly frozen, and then lyophilized overnight.

### 2.5. Protein Concentrations

To determine protein concentrations of −80 °C sEVs and lyophilized hASC sEVs, a nanodrop spectrophotometer was used. A 1 μL amount of sample was placed on the nanodrop and measured at an absorbance of 280 nm. Measurements were conducted thrice for each sample.

### 2.6. Scratch Assay

To evaluate the hASC sEVs for wound-healing efficacy, a scratch assay was performed using HT22 cells. HT22 cells were grown to confluency in a 12-well plate, and a scratch was created using a p10 pipet tip. The CM was replaced and 2 μg/mL of hASC sEVs or lyophilized hASC sEVs was added to the cells. Cell migration was imaged on a Keyence BZ-X810 microscope from Keyence Corporation, Tampa, FL, 33607) at 0, 16, and 24 h at 4× magnification.

### 2.7. DiD-Labeled sEV Uptake

HT22 cells were grown to confluency in an 8-well chamber plate and used to assess exosomal uptake. The hASC sEVs were incubated with 100 nM DiD, prepared according to manufacturer’s instructions in EtOH, for 30 min. Then, 2 μg/mL of labeled hASC sEVs were added to HT22 cells stained with 4′,6-diamidino-2-phenylindole (DAPI) and imaged using a Keyence BZ-X810 microscope after 24 h at 40× magnification. At the 24 h mark, the fluorescence intensity was measured and assessed using the Keyence software (v 1.1.1.8), Keyence Corporation, Tampa, FL, 33607. One window was used to measure the fluorescence intensity. A window was chosen if it had (1) a high concentration of sEVs present and (2) a wide spread of cells present with little to no clumping.

### 2.8. In Vitro Inflammation Model

HT22 cells were plated in an 8-well chamber plate and grown to confluency. Cells were treated with 5 ng/mL of LPS for six hours. Medium was changed, and 2 μg/mL of sEVs was added for 18 h. Immunochemistry was performed as described below.

### 2.9. In Vitro Oxidative Stress Model

HT22 cells were plated in an 8-well chamber plate and grown to confluency. Cells were treated with 1:1000 H_2_O_2_ for 1 h. Medium was changed, and 2 μg/mL of sEVs was added for 18 h. Immunochemistry was performed as described below.

### 2.10. Immunochemistry

HT22 cells were plated in an 8-well chamber plate and grown to confluency. Medium was then removed, and cells were washed 3 times with PBS and fixed with 4% paraformaldehyde for 30 min. Cells were rinsed with PBS and blocked with 1% bovine serum albumin for 30 min at room temperature. Cells were washed 3 times with PBS and then incubated with primary antibodies for Ki-67 overnight at 4 °C. Cells were rinsed with PBS 3 times and incubated in secondary fluorescent antibody for 1 h at room temperature. Cells were stained with 4′,6-diamidino-2-phenylindole (DAPI) for 15 min at room temperature to visualize the nucleus. Samples were imaged at 20× using a Keyence microscope.

### 2.11. Statistical Analysis Methods

Statistical analyses were performed using GraphPad Prism SPSS Analysis Software V.10.0.2 (GraphPad PRISM™ Software (v 10.0.0), IBM SPSS Inc., Boston, MA, USA). Comparisons of the means were made between groups using unpaired Student’s *t*-tests or ANOVA analysis. The significance of the results is indicated by the (*) symbol with the corresponding *p*-values: * *p* < 0.05, ** *p* < 0.01, *** *p* < 0.001, **** *p* < 0.0001.

## 3. Results

### 3.1. Optimal Method of hASC sEV Preparation

**Figure 2:**
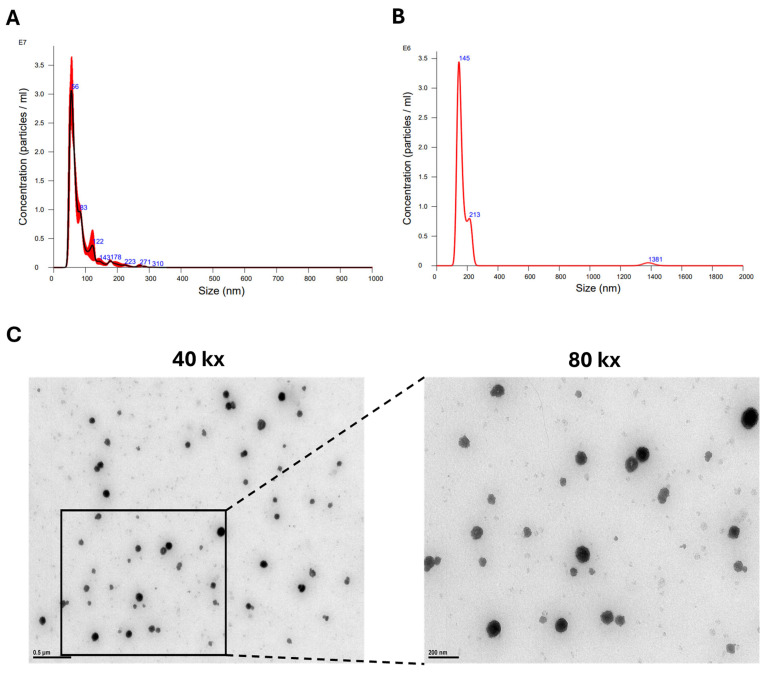
NTA and TEM data showing the sizes and concentrations of sEVs isolated from hASCs. (**A**) sEV elution from ExoSpin column showing an average size of 80.3 nm and concentration of 9.84 × 10^8^ particles/mL. (**B**) sEV elution from qEV column showing a peak size of 145 nm and concentration of 9.20 × 10^7^ particles/mL. Measurements in triplicate for each sample. (**C**) TEM image showing hASC sEVs extracted from a qEV column at 40 k× and 80 k× magnification. Scale bar = 500 nm and 200 nm, respectively.

Kits using reagents to precipitate EVs from hASC conditioned media (CM) sometimes result in impurities in the final sEV preparation. Hence, we sought to compare two widely used methods to determine the method yielding pure sEVs (size 35–200 nm). The hASC cells were plated in T-25 flasks, and conditioned media (CM) was collected. The sEVs were isolated from the CM as described in the Methods section. The NTA data revealed that the qEV columns demonstrated cleaner particle size distribution peaks compared with the ExoSpin columns. The particle size distribution peak from the ExoSpin column had a sharp peak at 56 nm, but the tailing of the peak showed various other sizes (Figure 2A). The peak from the qEV isolation showed a sharp peak at 145 nm and one smaller peak at 213 nm (Figure 2B). These data established that the qEV column combined with ultrafiltration produces pure particle size distributions compared with the ExoSpin Columns. From here on, the qEV method was used to prepare and purify the hASC sEVs for the experiments. To establish the morphology of the hASC sEVs, transmission electron microscopy was used to image the sEVs after extraction (Figure 2C). The TEM images show round-shaped sEVs at a size of 141 nm at 40 k× and 80 k× magnification.

### 3.2. Lyophilization Has No Effect on Concentration or Morphology of hASC sEVs

The hASC sEVs were purified using qEV columns and divided into two aliquots. One aliquot was stored at −80 °C, and the other aliquot was lyophilized. The hASC sEVs were lyophilized, stored at room temperature (RT), and analyzed after 2 weeks and compared with the sEVs stored at −80 °C. At the 2-week mark, the −80 °C stored exosome and the lyophilized RT samples with trehalose had protein concentrations of approximately 2 μg/mL. This was also observed at the 1- and 2-month marks. All protein concentrations were within 1% of each other (Table 1) between the −80 °C stored sEVs and the lyophilized RT samples with trehalose (Table 1). For lyophilized sEVs without trehalose, there was a decrease in protein concentration of almost 50% seen at the 2-week mark.

**Figure 3:**
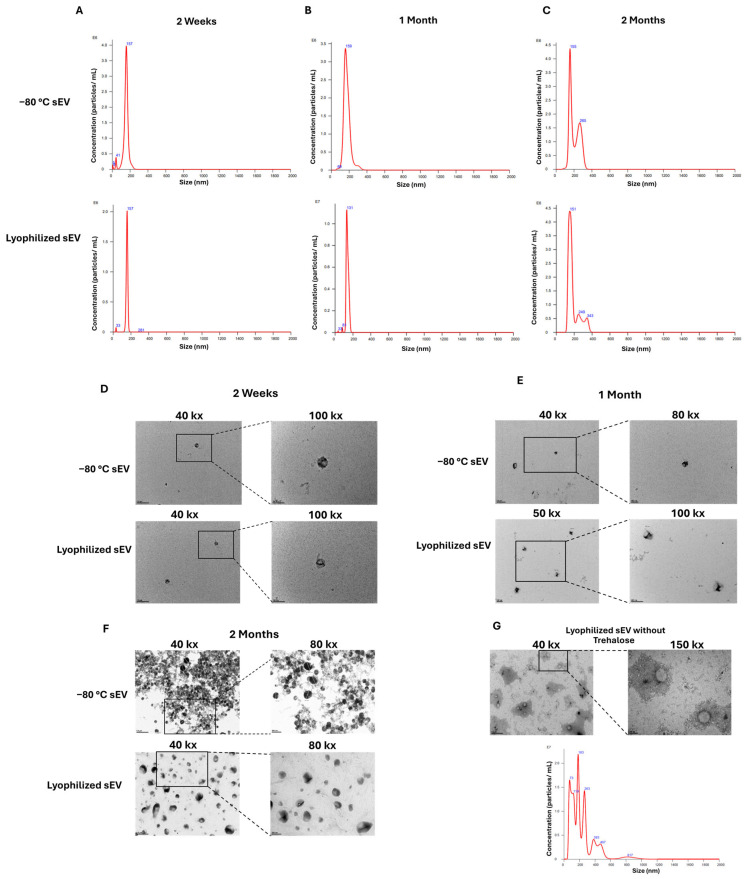
TEM images of −80 °C sEVs and lyophilized sEVs at low and high magnifications and NTA data showing the sizes of the −80 °C sEVs and lyophilized sEVs after 2 weeks, 1 month, and 2 months. (**A**) NTA data showing sizes of hASC sEVs after 2 weeks of storage. (**B**) NTA data showing sizes of hASC sEVs after 1 month of storage. (**C**) NTA data showing sizes of hASC sEVs after 2 months of storage. (**D**) TEM images of sEVs after 2 weeks of storage. (**E**) TEM images of sEVs after 1 month of storage. (**F**) TEM images of sEVs after 2 months of storage. (**G**) NTA data and TEMs of lyophilized sEVs without trehalose after 2 weeks of storage. For 40 k× images, the scale bar = 500 nm. For 50 k×, 80 k×, and 100 k× images, the scale bar = 200 nm. For 150 k× images, the scale bar = 100 nm.

To evaluate the morphology, hASC sEVs were imaged using TEM. At two weeks, both the −80 °C sEVs and the lyophilized RT with trehalose sEVs showed intact spherical morphology shapes (Figure 3D). The sizes of the sEVs were within 10% compared with the freshly extracted sEVs based on NTA data (Figure 3A). The lyophilized RT sEVs without trehalose did not show intact morphology, and a change in size was observed based on TEM images and NTA data (Figure 3G). There were also signs of aggregation and loss of protein observed. Due to these changes, lyophilized sEVs without trehalose were not evaluated after 2 weeks. The sizes of the sEV samples at 1 and 2 months, stored either at −80 °C or lyophilized and stored at room temperature, remained within 10% of each other (Figure 3B,C). There were no changes observed in morphology after 1 month in both the sEVs stored at −80 °C and sEVs lyophilized and stored at RT. After 2 months of storage, the morphology of the sEV samples was still spherical for both lyophilized sEVs stored at room temperature and sEVs stored at −80 °C (Figure 3E,F). However, samples stored at −80 °C showed signs of aggregation in the TEM images and NTA data. Loss of protein was also observed in the TEM images for the −80 °C sEVs.

### 3.3. Lyophilized sEVs Stored at Room Temperature Promote Wound Healing in Neuronal Cells

**Figure 4:**
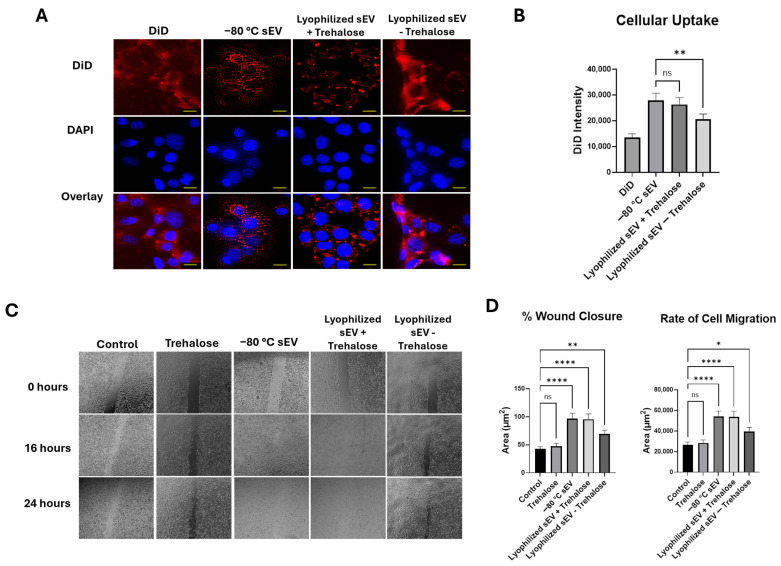
Cellular uptake and wound closure comparison of −80 °C sEVs and lyophilized RT sEVs at two weeks in HT22 cells. (**A**) Cellular uptake of DiD and DiD-labeled sEVs over 24 h, imaged at 40× (scale bar = 25 µm). (**B**) Graphical comparison of DiD intensity. The images for DiD, DAPI, and the overlay are all images of the same section of cells. DiD is being used to stain the sEVs in this study, while DAPI stains the nuclei of the cells. The overlay shows both DiD and DAPI in one image together to visualize how well the DiD-labeled sEVs are taken up by the cells. A high cell colocalization with sEV indicates that a higher number of sEVs are making their way into the cells. Statistical analysis was performed by ANOVA analysis, with no significant differences between −80 °C stored sEVs compared with lyophilized RT sEVs with trehalose. (**C**) Scratch assay over 24 h, imaged at 4×. (**D**) Comparison of % wound closure and rate of cell migration between samples. The images in each column shown in the figure demonstrate a scratch in the HT22 cells at different time intervals. The scratch shown at each time interval is the same scratch in the same section of cells to demonstrate how each treatment affects the ability of the cells to close the wound gap. A high % wound closure indicates how well the sEVs are repairing the damage to the cells. Statistical analysis was performed by ANOVA analysis (* *p* < 0.05, ** *p* < 0.01, and **** *p* ≤ 0.0001, no significance (ns)).

To assess cellular uptake of the hASC sEVs in HT22 cells, 2 μg/mL of DiD-labeled sEVs were applied to the cells and monitored for 24 h. The sEVs were incubated in DiD at a concentration of 100 nM for 30 min and centrifuged to remove excess dye, and then added to the cells. After 24 h, DAPI stain was added and used to visualize the nuclei of the cells in this experiment. We first evaluated the staining effects of DiD alone on the cells. DiD without the sEVs stained the cell membrane of the cells, whereas the labeled sEVs can be seen as small circles in the nucleus and cytoplasm. The fluorescence intensity of the DiD was about 17,000 (Figure 4A). Both −80 °C stored sEVs and lyophilized hASC sEVs with trehalose were taken up efficiently by the HT22 cells. The fluorescence intensity for the −80 °C stored sEVs and lyophilized RT sEVs was about 27,000 and 26,000, respectively; however, for the lyophilized sEVs without trehalose, the intensity was about 20,000 (Figure 4B).

We then evaluated the efficacy of the lyophilized hASC sEVs for treating wounds using the in vitro scratch assay, which creates cell-free gaps in the cell culture mimicking wounds. HT22 cells were wounded by creating a scratch (described in Methods). Then, 2 μg/mL of sEVs stored at −80 °C and a lyophilized sEV sample stored at room temperature was applied to the HT22 cells. Wound closure was assessed for 24 h and compared with a control without sEVs (Figure 4C). The wound closure percentages of the −80 °C stored sEVs and the lyophilized RT sEVs with trehalose at the 24 h mark were comparable, at 95% for both samples. The lyophilized RT sEVs without trehalose showed a wound closure of only 68%. Due to this low wound closure percentage and uptake, lyophilized sEVs without trehalose were not used in further studies. The rate of migration of HT22 cells to close the wound was also similar between the −80 °C stored sEVs and the lyophilized RT sEVs with trehalose (Figure 4D). We also assessed the effects of trehalose on its own in the cells. A wound closure of 59% was observed, a 5% difference from the control.

### 3.4. Lyophilized sEVs Promote Wound Healing Efficiently After 1 Month at Room Temperature

**Figure 5:**
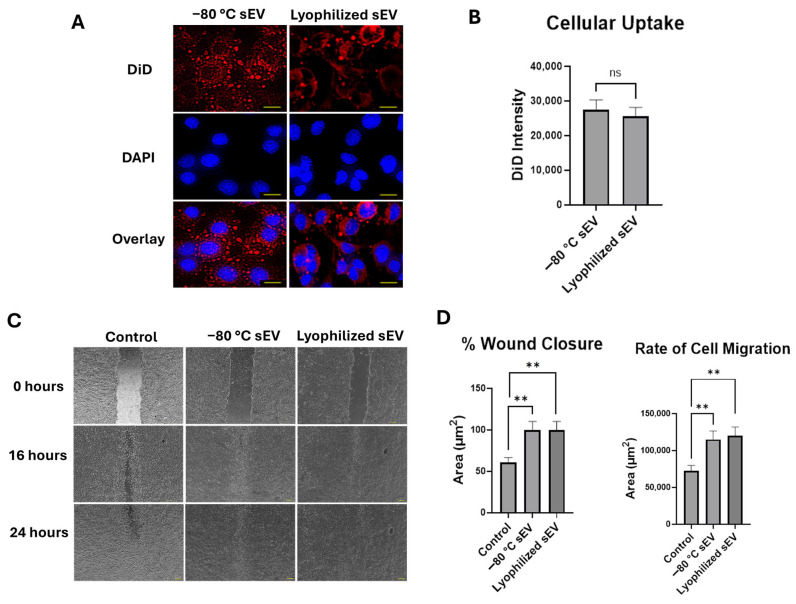
Cellular uptake and wound closure comparison of −80 °C sEVs and lyophilized RT sEVs at one month in HT22 cells. (**A**) Cellular uptake of DiD-labeled sEVs over 24 h, imaged at 40× (scale bar = 25 µm). (**B**) Graphical comparison of DiD intensity. The images for DiD, DAPI, and the overlay are all images of the same section of cells. DiD is being used to stain the sEVs in this study, while DAPI stains the nuclei of the cells. The overlay shows both DiD and DAPI in one image together to help visualize how well the DiD-labeled sEVs are taken up by the cells. A high cell colocalization with sEVs indicates that a higher number of sEVs are making their way into the cells. Statistical analysis was performed by two-tailed Student’s *t*-test, no significance (ns). (**C**) Scratch assay over 24 h, imaged at 4×. (**D**) Comparison of % wound closure and rate of cell migration between samples. The images in each column shown in the figure demonstrate a scratch in the HT22 cells at different time intervals. The scratch shown at each time interval is the same scratch in the same section of cells to demonstrate how each treatment affects the ability of the cells to close the wound gap. A high % wound closure indicates how well the sEVs are repairing the damage to the cells. Statistical analysis was performed by ANOVA analysis ** *p* < 0.01).

The cellular uptake was also assessed for 1 month, and no significant changes were observed between the lyophilized RT and the −80 °C stored sEVs (Figure 5A). The fluorescence intensities for the −80 °C stored sEVs and the lyophilized RT sEVs were about 27,000 and 25,000, respectively (Figure 5B). Next, we evaluated the efficacy of the lyophilized hASC sEVs stored at room temperature for one month. Scratch assay was performed in HT22 cells followed by treatment with −80 °C stored sEVs and lyophilized (room temperature for 1 month) hASC sEVs (Figure 5C). There was no change in the efficacy of the lyophilized RT sEVs. Both the −80 °C sEVs and the lyophilized RT samples showed a 100% wound closure at 24 h. The wound treated with lyophilized, RT sEVs had a higher rate of cell migration to fill the wound gap (Figure 5D).

### 3.5. Lyophilized sEVs Promote Wound Healing Efficiently After 2 Months at Room Temperature

**Figure 6:**
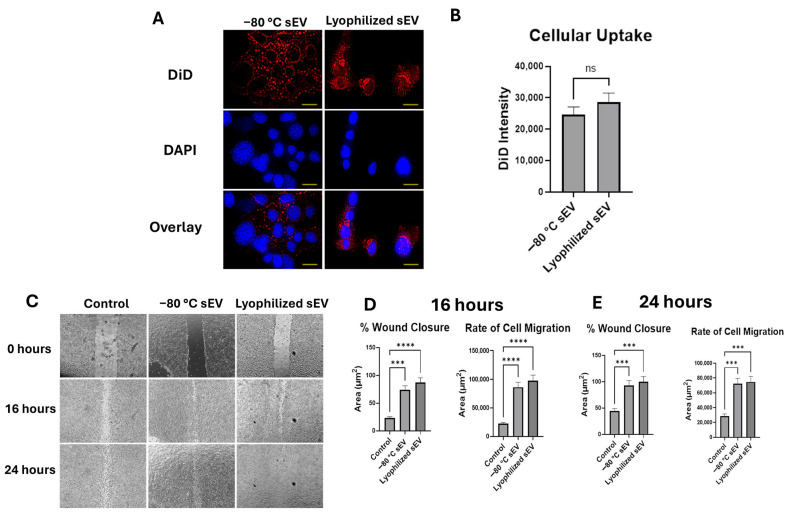
Cellular uptake and wound closure comparison of −80 °C sEVs and lyophilized RT sEVs at two months in HT22 cells. (**A**) Cellular uptake of DiD-labeled sEVs over 24 h, imaged at 40× (scale bar = 25 µm). (**B**) Graphical comparison of DiD intensity. The images for DiD, DAPI, and the overlay are all images of the same section of cells. DiD is being used to stain the sEVs in this study, while DAPI stains the nuclei of the cells. The overlay shows both DiD and DAPI in one image together to help visualize how well the DiD-labeled sEVs are taken up by the cells. A high cell colocalization with sEV indicates that a higher number of sEVs are making their way into the cells. Statistical analysis was performed by two-tailed Student’s *t*-test, no significance (ns). (**C**) Scratch assay over 24 h, imaged at 4×. (**D**) Comparison of % wound closure and rate of cell migration between samples after 16 h. Statistical analysis was performed by two-tailed Student’s *t*-test. (**E**) Comparison of % wound closure and rate of cell migration between samples after 24 h. The images in each column shown in the figure demonstrate a scratch in the HT22 cells at different time intervals. The scratch shown at each time interval is the same scratch in the same section of cells to demonstrate how each treatment affects the ability of the cells to close the wound gap. A high % wound closure indicates how well the sEVs are repairing the damage to the cells. Statistical analysis was performed by ANOVA analysis *** *p* < 0.001, and **** *p* ≤ 0.0001.

Cellular uptake and wound healing were assessed after 2 months of storage at room temperature following lyophilization. Both samples of sEVs displayed similar DiD intensity values over a 24 h period (Figure 6A,B). We then investigated the efficacy of the lyophilized hASC sEVs (stored at room temperature for 2 months) in HT22 cells using a scratch assay over 24 h (Figure 6C). At the 16 h mark, the −80 °C sEVs showed a significant difference in wound closure compared with the lyophilized RT sEVs. The −80 °C sEVs were at 74%, while the lyophilized sEVs were at 87% wound closure (Figure 6D). The lyophilized RT sEVs also demonstrated better efficacy, showing a wound closure of 100%, compared with the −80 °C sEVs, which showed a closure of 92% at the 24 h mark (Figure 6E).

### 3.6. Lyophilized sEVs Rescue Cell Proliferation in Neuronal Cells with Underlying Inflammation In Vitro

**Figure 7:**
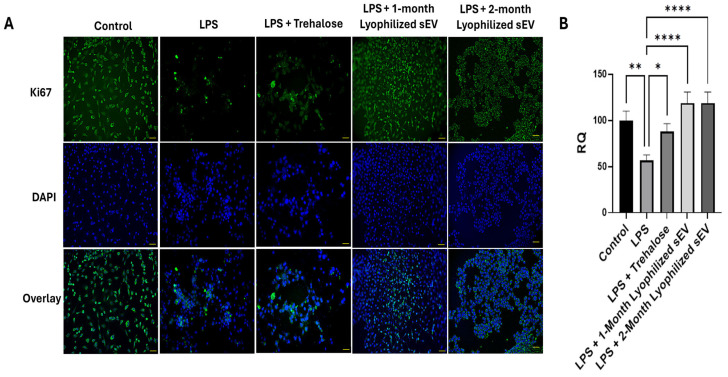
Immunochemistry assay of HT22 cells treated with 5 ng/mL LPS for 6 h, after which the medium was changed, and the cells were treated with 2 µg of sEVs for 18 h. (**A**) Cells were stained using Ki-67 as a marker for proliferation and DAPI as a nucleus stain and imaged with a Keyence BZx-810 microscope at 20× (scale bar = 50 µm). (**B**) Determination of colocalization of Ki-67 and DAPI was determined using the Keyence software. The images for Ki67, DAPI, and the overlay are all images of the same section of cells. Ki67 stains proliferating cells, while the DAPI stains the nuclei of the cells. The overlay shows both the Ki67 and DAPI in one image together to visualize cell proliferation occurring from each treatment. Statistical analysis was performed by ANOVA analysis (* *p* < 0.05, ** *p* < 0.01, and **** *p* ≤ 0.0001).

The efficacy of the lyophilized hASC sEVs was investigated using a cell proliferation assay in an inflammation model. HT22 cells were exposed to lipopolysaccharide (LPS) to mimic underlying inflammation and treated with 2 μg/mL lyophilized RT hASC sEVs (stored at room temperature for one or two months) for 24 h. The cells were then stained with Ki-67, a marker for proliferation, and imaged on a Keyence microscope (Figure 7A,B). After exposure to LPS, cell proliferation decreased dramatically, which was rescued by treatment with the lyophilized RT hASC sEVs.

After treatment with the hASC sEVs, proliferation increased, which is what was observed during this study. Cells without any treatment were used as a control, and the percent of Ki-67 was 51%. Cells that were treated with LPS showed a decrease in proliferation. Cells that were treated with LPS followed by lyophilized sEVs showed a rescue of cell proliferation. We also looked at the effects of trehalose and observed a slight increase in proliferation, but it was significantly lower compared with the rescue by sEVs.

### 3.7. Lyophilized sEVs Decrease Oxidative Stress in Neuronal Cells

**Figure 8:**
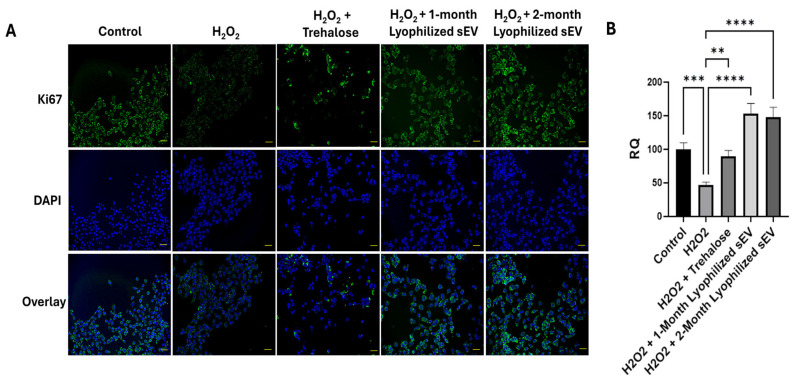
Immunochemistry assay of HT22 cells treated with 1:1000 hydrogen peroxide for 1 h, after which the medium was changed, and the cells were treated with 2 µg of lyophilized sEVs for 18 h. (**A**) Cells were stained using Ki-67 as a marker for proliferation and DAPI as a nucleus stain and imaged with a Keyence BZx-810 microscope at 20× (scale bar = 50 µm). (**B**) Determination of colocalization of Ki-67 and DAPI was determined using the Keyence software. The image for Ki67, DAPI, and the overlay are all images of the same section of cells. Ki67 stains proliferating cells, while the DAPI stains the nuclei of the cells. The overlay shows both the Ki67 and DAPI in one image together to visualize cell proliferation occurring from each treatment. Statistical analysis was performed by ANOVA analysis (** *p* < 0.01, *** *p* < 0.001, **** *p* ≤ 0.0001).

Neuronal cells were treated with hydrogen peroxide (H_2_O_2_) to mimic oxidative stress for one hour, and the efficacy of lyophilized sEVs for rescuing proliferation was assessed over 18 h (Figure 8A). After treatment with sEVs post oxidative stress, an increase in proliferation was observed in each type of sEV. Results showed an increase of 107% for one-month lyophilized samples and 102% for two-month lyophilized sEVs (Figure 8B). This demonstrates that the lyophilized sEVs, stored up to two months at room temperature, are efficient at decreasing oxidative stress in neuronal cells. Trehalose treatment did not rescue oxidative stress induced by peroxide.

## 4. Discussion

In this study, we have established that qEV columns are a better isolation method than ExoSpin kits. We also established that lyophilization with the addition of trehalose preserves the size and morphology of hASCs sEVs. We were then able to test if the lyophilized sEVs performed equally to the −80 °C sEVs after being stored at RT using several in vitro assays of neuronal injury that mimic TBI. We found that the lyophilized RT sEVs were significantly better at promoting wound healing than the −80 °C stored sEVs. This was demonstrated through physical injury, inflammation, and oxidative stress.

We previously demonstrated the efficacy of hASC sEVs in promoting repair and regeneration in traumatic brain injury in vivo [4,26,27]. The lyophilized hASC sEVs will be used in a similar setup. Other groups have also utilized sEVs derived from ASC and showed that they have immunoregulatory properties [28,29]. To address the limitations of room temperature storage and viability of hASC sEVs, we undertook this systematic evaluation. First, we evaluated the effect on the purity of sEVs using two different size exclusion techniques. Comparisons of size and concentration were evaluated using NTA data. In accordance with previous studies, the qEV size exclusion column coupled with ultrafiltration produced pure sEVs efficiently [30,31]. Our previous studies used CD9, CD63, and CD81 markers to verify that the sEVs described here contain exosomes [4,26,27].

The hASC sEVs have a tremendous potential in wound healing. However, storage remains an issue for translation to clinics. In this study, the use of lyophilization as a storage method for sEVs was explored. In previous studies, lyophilization has been investigated as a storage method for sEVs using trehalose. It was reported that sEVs can be stored at room temperature for one week after lyophilization with the addition of trehalose [21]. Without trehalose, aggregation and a change in morphology can occur, which we have demonstrated. Here, we sought to evaluate the efficacy of lyophilized hASC sEVs on neuronal wound healing potential after storage at room temperature for two months, which has not been demonstrated so far. To evaluate the effects of lyophilization, the morphology, size, and functionality of the hASC sEVs were evaluated after two, four, and eight weeks of storage at room temperature. Our data demonstrate that lyophilization is an effective long-term storage method, and we further establish that the protein concentration and size were unchanged between the lyophilized samples with trehalose. During the lyophilization process, trehalose can form a protective layer around the bilayer of the sEV. Trehalose can form hydrogen bond interactions with the polar head groups of the bilayer, effectively keeping the bilayer and cargo inside the sEVs safe from damage [32]. It was also demonstrated that the morphology and size of the lyophilized sEVs after two months did not show a difference between samples. It is also worth noting that the lyophilized samples showed a smaller size distribution than the sEVs stored at −80 °C.

Our results have demonstrated that sEVs are efficiently being taken up by HT22 cells. In some cases, aggregation of the sEVs can be seen, which could be due to their low zeta potential [33]. We have initiated studies to investigate the mechanism underlying the aggregation of the sEVs. It is reported that smaller extracellular vesicles such as sEVs have faster cellular uptake [34]. The uptake demonstrated by each sEV sample in this study supports this notion, as each sample is relatively in the same size range. Our results also demonstrate that the sEVs are present in the cytoplasm and nucleus of the HT22 cells, showing efficient internalization of sEVs. There was also no change observed in the efficiency of the sEVs’ cellular uptake post lyophilization. hASC sEVs have shown regenerative capabilities due to the biological material secreted inside of their sEVs. We have previously shown that these hASC sEVs can be used for wound healing in vitro and in vivo in TBI models [4,26,27].

Finally, we also evaluated the effects of lyophilization on the efficacy of hASC sEVs using in vitro neuronal injury models mimicking physical injury, inflammation, and oxidative stress. We have shown that hASC sEVs have regenerative capabilities and can be used to increase cell proliferation in neuronal cells in vitro [26]. In this study, we used lyophilized sEVs to assess the efficacy of the lyophilization of sEVs stored at room temperature, and the results demonstrate that the efficacy of sEVs to promote wound healing is maintained. TBI is an injury that affects many people every year, and it is known to cause secondary injuries that damage neuronal cells. LPS, which was used to mimic underlying inflammation caused by TBI in vitro, and hydrogen peroxide, which mimics oxidative stress caused by TBI, were used to assess the lyophilized sEVs’ efficacy to rescue the cell proliferation. It was also important to evaluate the effects of trehalose under oxidative stress, as it has been reported that trehalose can increase proliferation under oxidative stress in high concentrations. These data further support the assertion that lyophilization can be used as a storage method for sEVs at room temperature for two months and can be effectively used to treat neuronal injury seen in TBI.

The lyophilization of sEVs could also be applied to engineered EVs. EVs have regularly been used as therapeutics in the past few years by loading them with mRNA, proteins, peptides, and more. Long-term storage of the engineered vesicles would still require storage temperatures of −20 °C or −80 °C, causing a potential issue in clinical settings. Lyophilization could be proposed as a solution to this issue. In a previous study, sEVs loaded with Gaussia luciferase showed a little change in the pharmacokinetics of the engineered sEVs before and after lyophilization, suggesting that lyophilization can be used for engineered EVs [21]. To our knowledge, this is the only study that has shown lyophilization of engineered EVs. There is still much more research that should be performed regarding the lyophilization of engineered EVs.

In summary, we have demonstrated that lyophilization can be used as an effective room temperature storage of hASC sEVs for two months without any harmful effects on morphology, concentration, cell migration, and cellular uptake. We have also established that the efficacy of lyophilized sEVs for wound healing is comparable to sEVs stored at −80 °C up to 4 weeks, and is better than the sEVs stored at −80 °C after 8 weeks, based on in vitro assays used to mimic TBI injuries. We have initiated in vivo studies using lyophilized, room temperature stored sEVs in rodent models of TBI. In the previous study, we demonstrated the intranasal route of delivery of hASC sEVs post TBI, and we have undertaken a similar intranasal delivery of lyophilized sEVs. The in vivo study will provide information on genomic changes by RNAseq, which can be compared with our previously published results using freshly prepared sEVs. Here, we successfully demonstrated the viability and efficacy of lyophilized sEVs stored at room temperature, thereby enabling the initiation of in vivo studies using lyophilized RT sEVs. The hASC sEVs are applied topically for dermal wounds [35] or administered intranasally for TBI [27], and they do not need any carrier, thus adding to the advantage of hASC sEVs in accelerating the healing of wounds.

## 5. Conclusions

In conclusion, we have shown that qEV produces clean size-distribution peaks. The sEVs extracted from qEV columns showed cleaner peaks based on the NTA results. Lyophilization can be used as a storage method for sEVs at room temperature for two months with the addition of trehalose without any damage to performance or structure. This method does not inhibit the performance of the hASC sEVs, as demonstrated by neuronal in vitro injury models such as scratch assay, LPS-induced inflammation, and hydrogen peroxide-induced oxidative stress. Thus, lyophilized sEVs stored at room temperature can be incorporated into first-aid kits in the future, which will advance their translation to clinic and routine use by all individuals.

## Figures and Tables

**Table 1:** Protein and particle concentrations of −80 °C sEVs and lyophilized room temperature sEVs. All measurements were performed in triplicate. Statistical analysis was performed by two-tailed Student’s *t*-test or ANOVA analysis.

Storage Time	Sample	Protein Concentration (μg/mL)	*p*-Value	Size (nm)	*p*-Value	Concentration (particles/mL)	*p*-Value
2 Weeks	−80 °C sEV	2.047 ± 0.036	0.0014	157 ± 32	0.1840	2.59 × 10^7^	
	Lyophilized sEV + trehalose	2.039 ± 0.037		157 ± 17		2.42 × 10^7^	0.2535
	Lyophilized sEV—trehalose	1.163 ± 0.027		182 ± 159		1.74 × 10^6^	
1 Month	−80 °C sEV	2.122 ± 0.015	0.8468	159 ± 39	0.0782	5.03 × 10^7^	0.1454
	Lyophilized sEV	2.158 ± 0.025		131 ± 23		1.44 × 10^7^	
2 Months	−80 °C sEV	2.003 ± 0.024	0.9135	155 ± 56	0.7649	2.12 × 10^7^	0.3788
	Lyophilized sEV	2.022 ± 0.017		151 ± 64		1.03 × 10^7^	

## Data Availability

The original contributions presented in the study are included in the article; further inquiries can be directed to the corresponding author.

## Supplementary Materials

The following supporting information can be downloaded at: https://www.mdpi.com/article/10.3390/biomedicines13020275/s1. Table S1: The raw data for Figure 4, Figure 5, Figure 6, Figure 7 and Figure 8; File S1: The original images for Figure 4, Figure 5, Figure 6, Figure 7 and Figure 8; File S2: BioRender publication permission for schematic of lyophilization process; File S3: BioRender publication permission for schematic of graphical abstract.

## References

[1] Mollayeva T., Mollayeva S., Colantonio A. (2018). Traumatic brain injury: Sex, gender and intersecting vulnerabilities. Nat. Rev. Neurol..

[2] McKee A.C., Daneshvar D.H. (2015). The neuropathology of traumatic brain injury. Handbook of Clinical Neurology.

[3] Acosta S.A., Tajiri N., Shinozuka K., Ishikawa H., Grimmig B., Diamond D.M., Sanberg P.R., Bickford P.C., Kaneko Y., Borlongan C.V. (2013). Long-term upregulation of inflammation and suppression of cell proliferation in the brain of adult rats exposed to traumatic brain injury using the controlled cortical impact model. PLoS ONE.

[4] Patel N.A., Moss L.D., Lee J.Y., Tajiri N., Acosta S., Hudson C., Parag S., Cooper D.R., Borlongan C.V., Bickford P.C. (2018). Long noncoding RNA MALAT1 in exosomes drives regenerative function and modulates inflammation-linked networks following traumatic brain injury. J. Neuroinflamm..

[5] Brett B.L., Gardner R.C., Godbout J., Dams-O’Connor K., Keene C.D. (2022). Traumatic Brain Injury and Risk of Neurodegenerative Disorder. Biol. Psychiatry.

[6] Dams-O’Connor K., Juengst S.B., Bogner J., Chiaravalloti N.D., Corrigan J.D., Giacino J.T., Harrison-Felix C.L., Hoffman J.M., Ketchum J.M., Lequerica A.H. (2023). Traumatic brain injury as a chronic disease: Insights from the United States Traumatic Brain Injury Model Systems Research Program. Lancet Neurol..

[7] Jiang J.Y., Gao G.Y., Feng J.F., Mao Q., Chen L.G., Yang X.F., Liu J.F., Wang Y.H., Qiu B.H., Huang X.J. (2019). Traumatic brain injury in China. Lancet Neurol..

[8] Elder G.A. (2015). Update on TBI and Cognitive Impairment in Military Veterans. Curr. Neurol. Neurosci. Rep..

[9] Lazarus R., Helmick K., Malik S., Gregory E., Agimi Y., Marion D. (2018). Continuum of the United States military’s traumatic brain injury care: Adjusting to the changing battlefield. Neurosurg. Focus..

[10] Yamamoto S., DeWitt D.S., Prough D.S. (2018). Impact & Blast Traumatic Brain Injury: Implications for Therapy. Molecules.

[11] Welsh J.A., Goberdhan D.C.I., O’Driscoll L., Buzas E.I., Blenkiron C., Bussolati B., Cai H., Di Vizio D., Driedonks T.A.P., Erdbrugger U. (2024). Minimal information for studies of extracellular vesicles (MISEV2023): From basic to advanced approaches. J. Extracell. Vesicles.

[12] Syzdykbayev M., Kazymov M., Aubakirov M., Kurmangazina A., Kairkhanov E., Kazangapov R., Bryzhakhina Z., Imangazinova S., Sheinin A. (2024). A Modern Approach to the Treatment of Traumatic Brain Injury. Medicines.

[13] Mathew B., Mansuri M.S., Williams K.R., Nairn A.C. (2021). Exosomes as Emerging Biomarker Tools in Neurodegenerative and Neuropsychiatric Disorders-A Proteomics Perspective. Brain Sci..

[14] Ha D., Yang N., Nadithe V. (2016). Exosomes as therapeutic drug carriers and delivery vehicles across biological membranes: Current perspectives and future challenges. Acta Pharm. Sin. B.

[15] Zhang H., Wang S., Sun M., Cui Y., Xing J., Teng L., Xi Z., Yang Z. (2022). Exosomes as smart drug delivery vehicles for cancer immunotherapy. Front. Immunol..

[16] Gao J., Li A., Hu J., Feng L., Liu L., Shen Z. (2022). Recent developments in isolating methods for exosomes. Front. Bioeng. Biotechnol..

[17] Langevin S.M., Kuhnell D., Orr-Asman M.A., Biesiada J., Zhang X., Medvedovic M., Thomas H.E. (2019). Balancing yield, purity and practicality: A modified differential ultracentrifugation protocol for efficient isolation of small extracellular vesicles from human serum. RNA Biol..

[18] Gamez-Valero A., Monguio-Tortajada M., Carreras-Planella L., Franquesa M., Beyer K., Borras F.E. (2016). Size-Exclusion Chromatography-based isolation minimally alters Extracellular Vesicles’ characteristics compared to precipitating agents. Sci. Rep..

[19] Jeyaram A., Jay S.M. (2017). Preservation and Storage Stability of Extracellular Vesicles for Therapeutic Applications. AAPS J..

[20] Kusuma G.D., Barabadi M., Tan J.L., Morton D.A.V., Frith J.E., Lim R. (2018). To Protect and to Preserve: Novel Preservation Strategies for Extracellular Vesicles. Front. Pharmacol..

[21] Charoenviriyakul C., Takahashi Y., Nishikawa M., Takakura Y. (2018). Preservation of exosomes at room temperature using lyophilization. Int. J. Pharm..

[22] Bahr M.M., Amer M.S., Abo-El-Sooud K., Abdallah A.N., El-Tookhy O.S. (2020). Preservation techniques of stem cells extracellular vesicles: A gate for manufacturing of clinical grade therapeutic extracellular vesicles and long-term clinical trials. Int. J. Vet. Sci. Med..

[23] Guan T., Miao Y., Xu L., Yang S., Wang J., He H., Tang X., Cai C., Xu H. (2011). Injectable nimodipine-loaded nanoliposomes: Preparation, lyophilization and characteristics. Int. J. Pharm..

[24] Kundu A.K., Chandra P.K., Hazari S., Ledet G., Pramar Y.V., Dash S., Mandal T.K. (2012). Stability of lyophilized siRNA nanosome formulations. Int. J. Pharm..

[25] Pascucci L., Scattini G. (2021). Imaging extracelluar vesicles by transmission electron microscopy: Coping with technical hurdles and morphological interpretation. Biochim. Biophys. Acta Gen. Subj..

[26] El Bassit G., Patel R.S., Carter G., Shibu V., Patel A.A., Song S., Murr M., Cooper D.R., Bickford P.C., Patel N.A. (2017). MALAT1 in Human Adipose Stem Cells Modulates Survival and Alternative Splicing of PKCdeltaII in HT22 Cells. Endocrinology.

[27] Moss L.D., Sode D., Patel R., Lui A., Hudson C., Patel N.A., Bickford P.C. (2021). Intranasal delivery of exosomes from human adipose derived stem cells at forty-eight hours post injury reduces motor and cognitive impairments following traumatic brain injury. Neurochem. Int..

[28] Chen Y., Li J., Ma B., Li N., Wang S., Sun Z., Xue C., Han Q., Wei J., Zhao R.C. (2020). MSC-derived exosomes promote recovery from traumatic brain injury via microglia/macrophages in rat. Aging.

[29] Tang L., Xu Y., Wang L., Pan J. (2023). Adipose-derived stem cell exosomes ameliorate traumatic brain injury through the NLRP3 signaling pathway. Neuroreport.

[30] Franco C., Ghirardello A., Bertazza L., Gasparotto M., Zanatta E., Iaccarino L., Valadi H., Doria A., Gatto M. (2023). Size-Exclusion Chromatography Combined with Ultrafiltration Efficiently Isolates Extracellular Vesicles from Human Blood Samples in Health and Disease. Int. J. Mol. Sci..

[31] Veerman R.E., Teeuwen L., Czarnewski P., Gucluler Akpinar G., Sandberg A., Cao X., Pernemalm M., Orre L.M., Gabrielsson S., Eldh M. (2021). Molecular evaluation of five different isolation methods for extracellular vesicles reveals different clinical applicability and subcellular origin. J. Extracell. Vesicles.

[32] Sivanantham A., Jin Y. (2022). Impact of Storage Conditions on EV Integrity/Surface Markers and Cargos. Life.

[33] Wang Y., Zhang L., Li Y., Chen L., Wang X., Guo W., Zhang X., Qin G., He S.H., Zimmerman A. (2015). Exosomes/microvesicles from induced pluripotent stem cells deliver cardioprotective miRNAs and prevent cardiomyocyte apoptosis in the ischemic myocardium. Int. J. Cardiol..

[34] Caponnetto F., Manini I., Skrap M., Palmai-Pallag T., Di Loreto C., Beltrami A.P., Cesselli D., Ferrari E. (2017). Size-dependent cellular uptake of exosomes. Nanomedicine.

[35] Patel R.S., Impreso S., Lui A., Vidyarthi G., Albear P., Patel N.A. (2022). Long Noncoding RNA GAS5 Contained in Exosomes Derived from Human Adipose Stem Cells Promotes Repair and Modulates Inflammation in a Chronic Dermal Wound Healing Model. Biology.

