# Supplementary material for: Lyophilized Small Extracellular Vesicles (sEVs) Derived from Human Adipose Stem Cells Maintain Efficacy to Promote Healing in Neuronal Injuries
Source: Biomedicines. 2025 Jan 23;13(2):275. doi: 10.3390/biomedicines13020275 (PMC11853053; doi:10.3390/biomedicines13020275)
Supplement: Supplementary file 1 [file biomedicines-13-00275-s001.zip › Supple S1 Original images for figure 2-8.pdf]

Figure 2

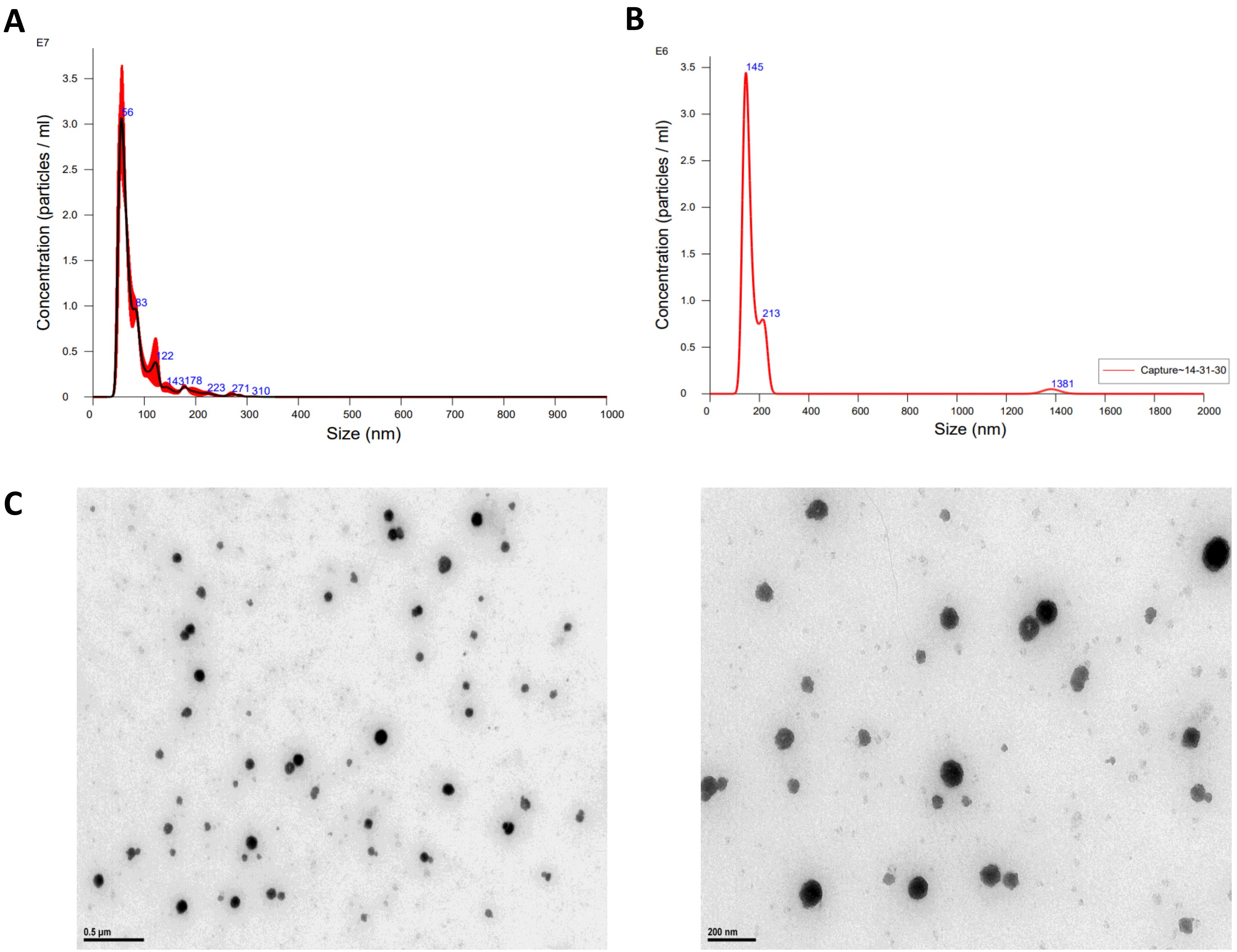
A
E7
3.5
3.0
2.5
2.0
1.5
1.0
0.5
0
Concentration (particles / ml)
56
83
122
143178
223
271
310
0
100
200
300
400
500
600
700
800
900
1000
Size (nm)
B
E6
145
213
1381
Capture~14-31-30
Concentration (particles / ml)
0
200
400
600
800
1000
1200
1400
1600
1800
2000
Size (nm)
C
0.5 µm
200 nm

Figure 3

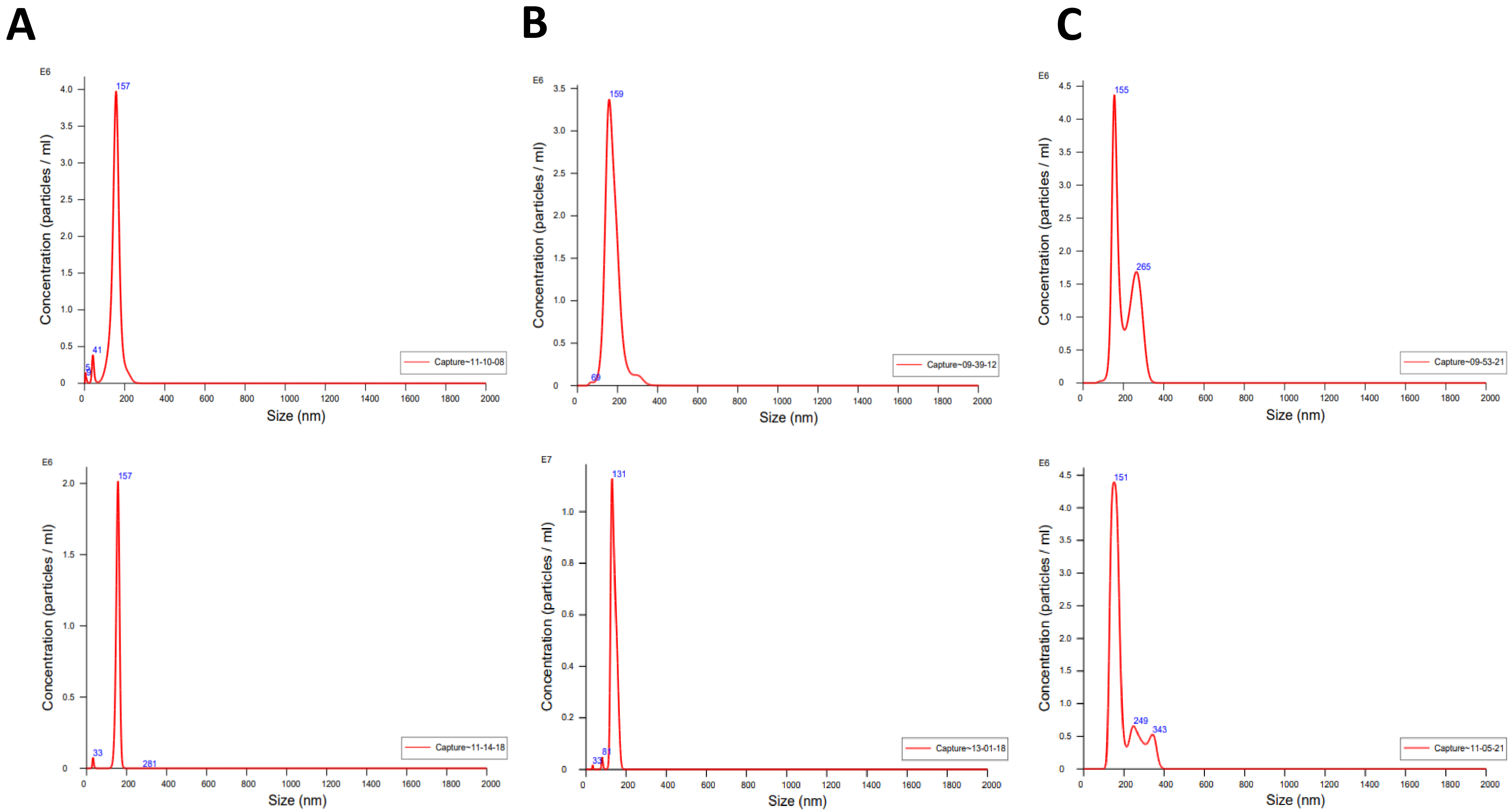
A
B
C
E6
Concentration (particles / ml)
Size (nm)
157
41
Capture~11-10-08
159
69
Capture~09-39-12
155
265
Capture~09-53-21
157
33
281
Capture~11-14-18
E7
131
33
81
Capture~13-01-18
151
249
343
Capture~11-05-21

Figure 3

D

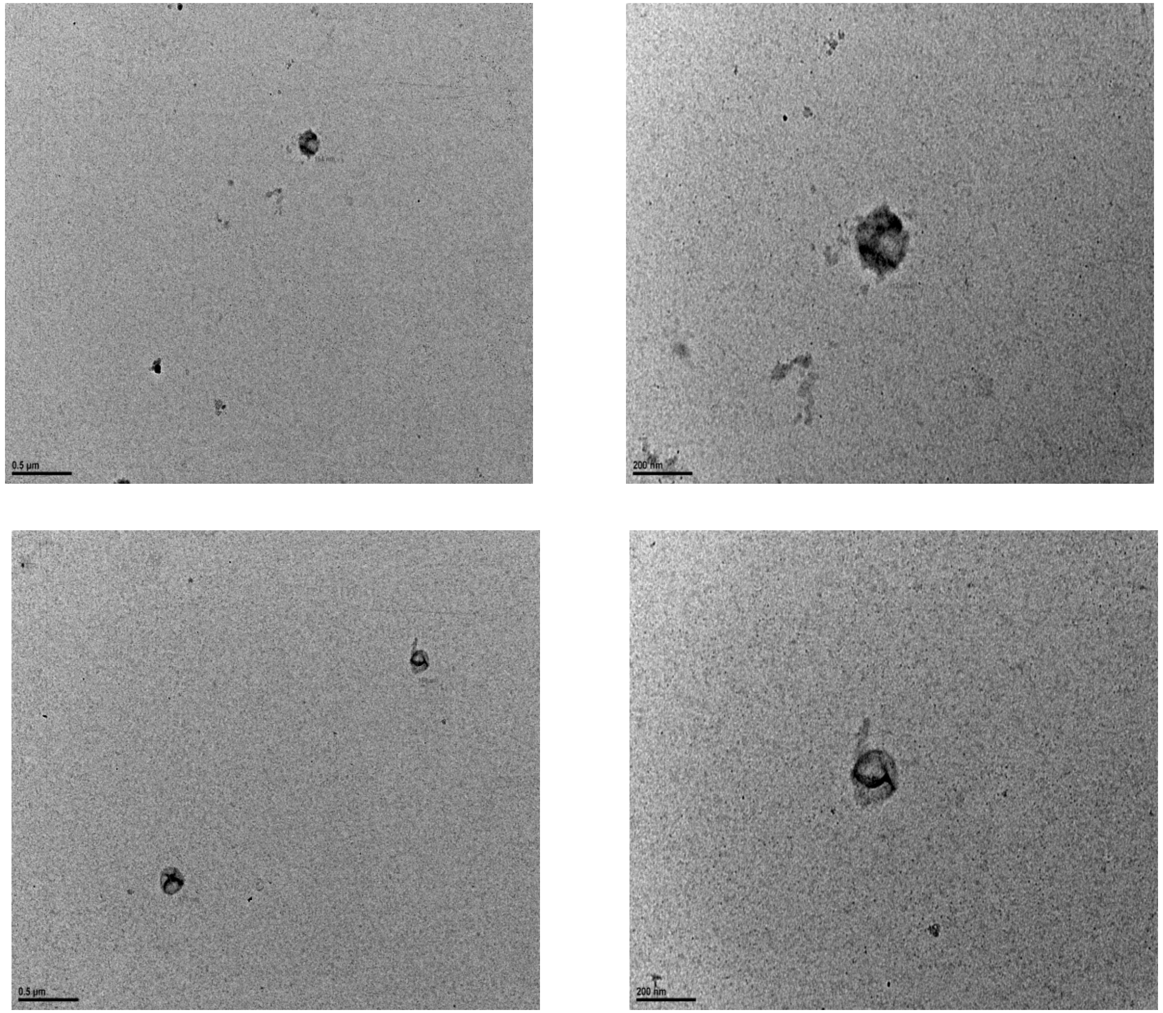

E

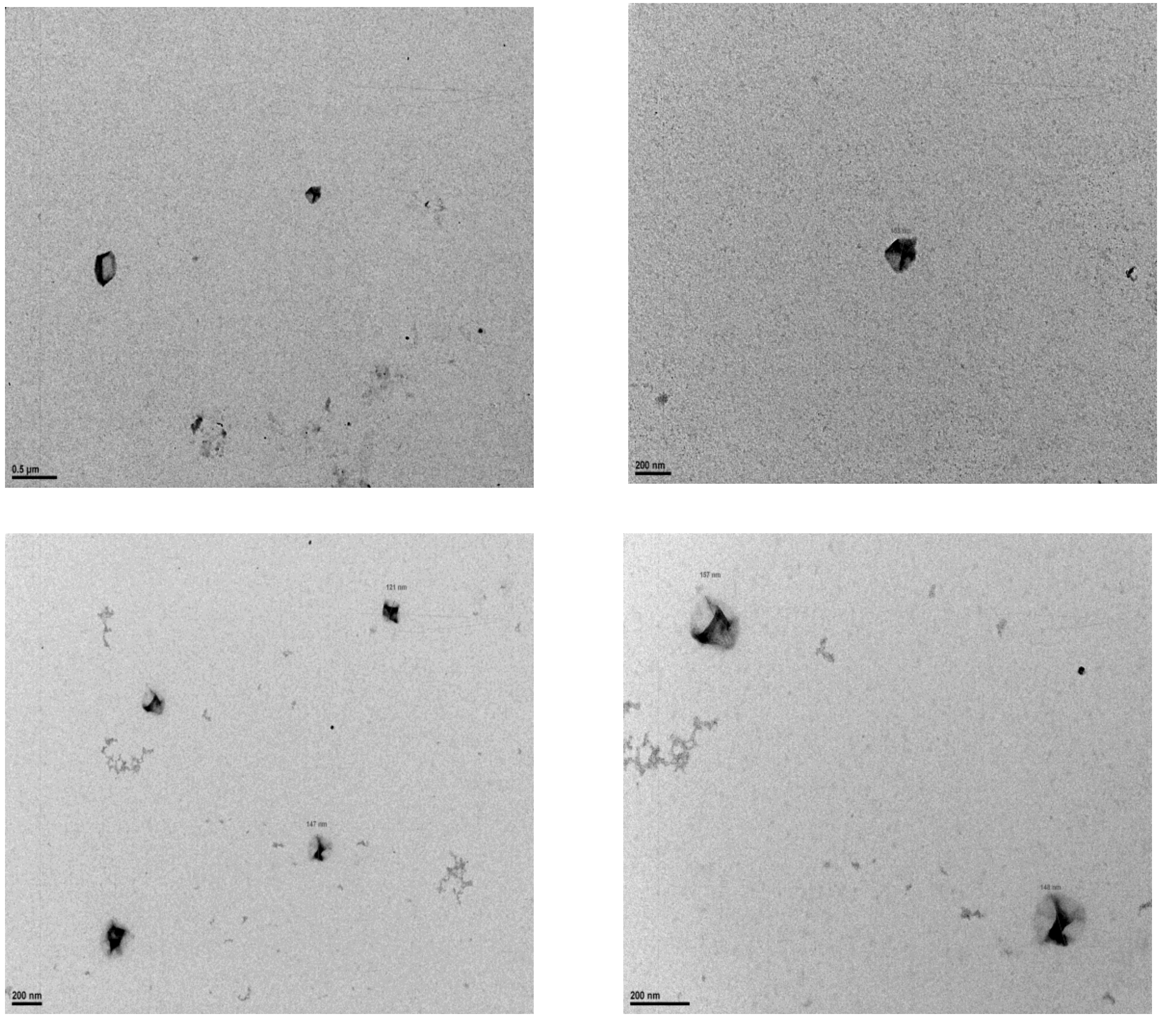

Figure 3

F

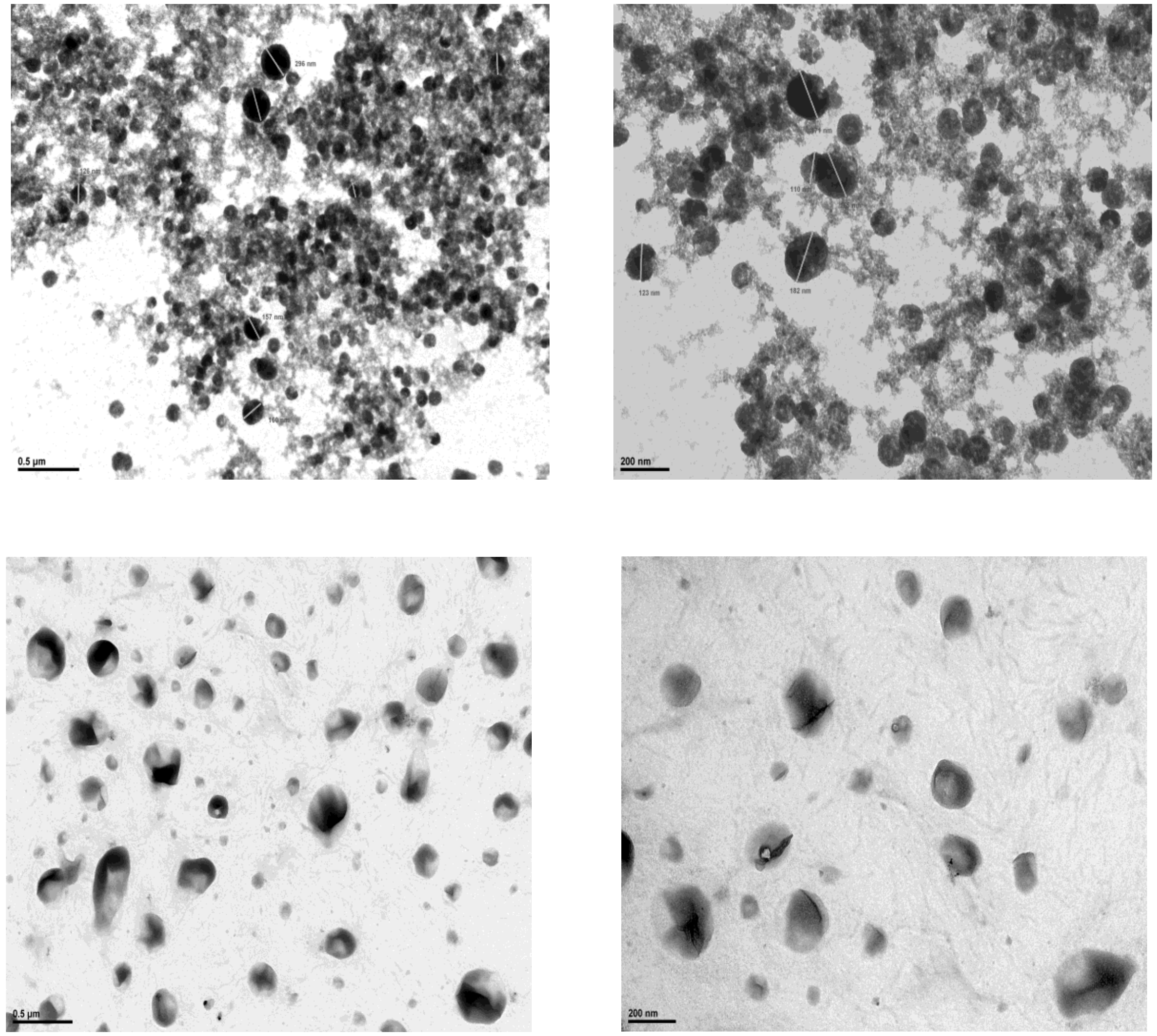
0.5 µm
200 nm
0.5 µm
200 nm

G

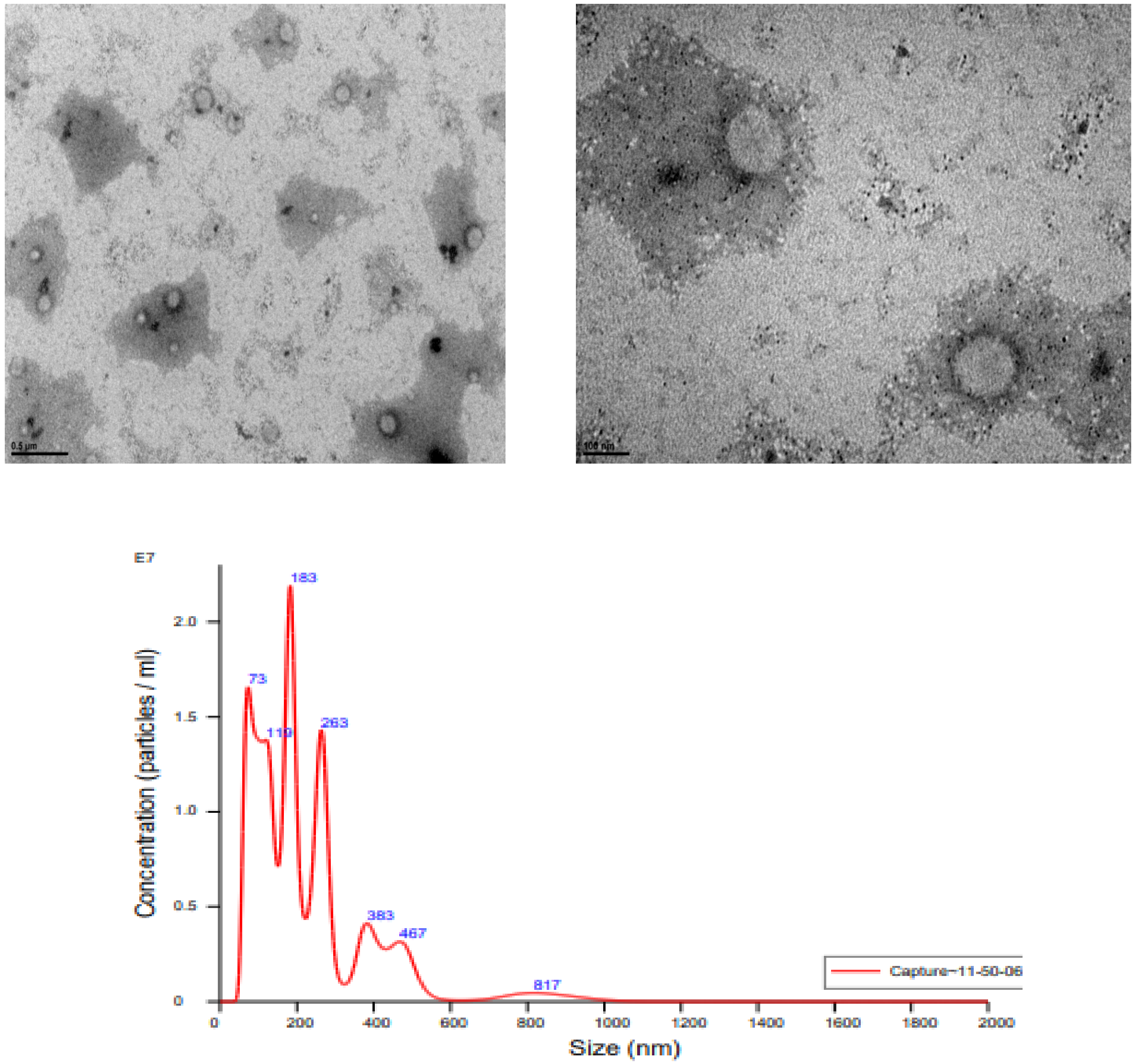
0.5 µm
100 nm
E7
Concentration (particles / ml)
2.0
1.5
1.0
0.5
0
73
119
183
263
383
467
817
0
200
400
600
800
1000
1200
1400
1600
1800
2000
Size (nm)
Capture-11-50-06

Figure 4

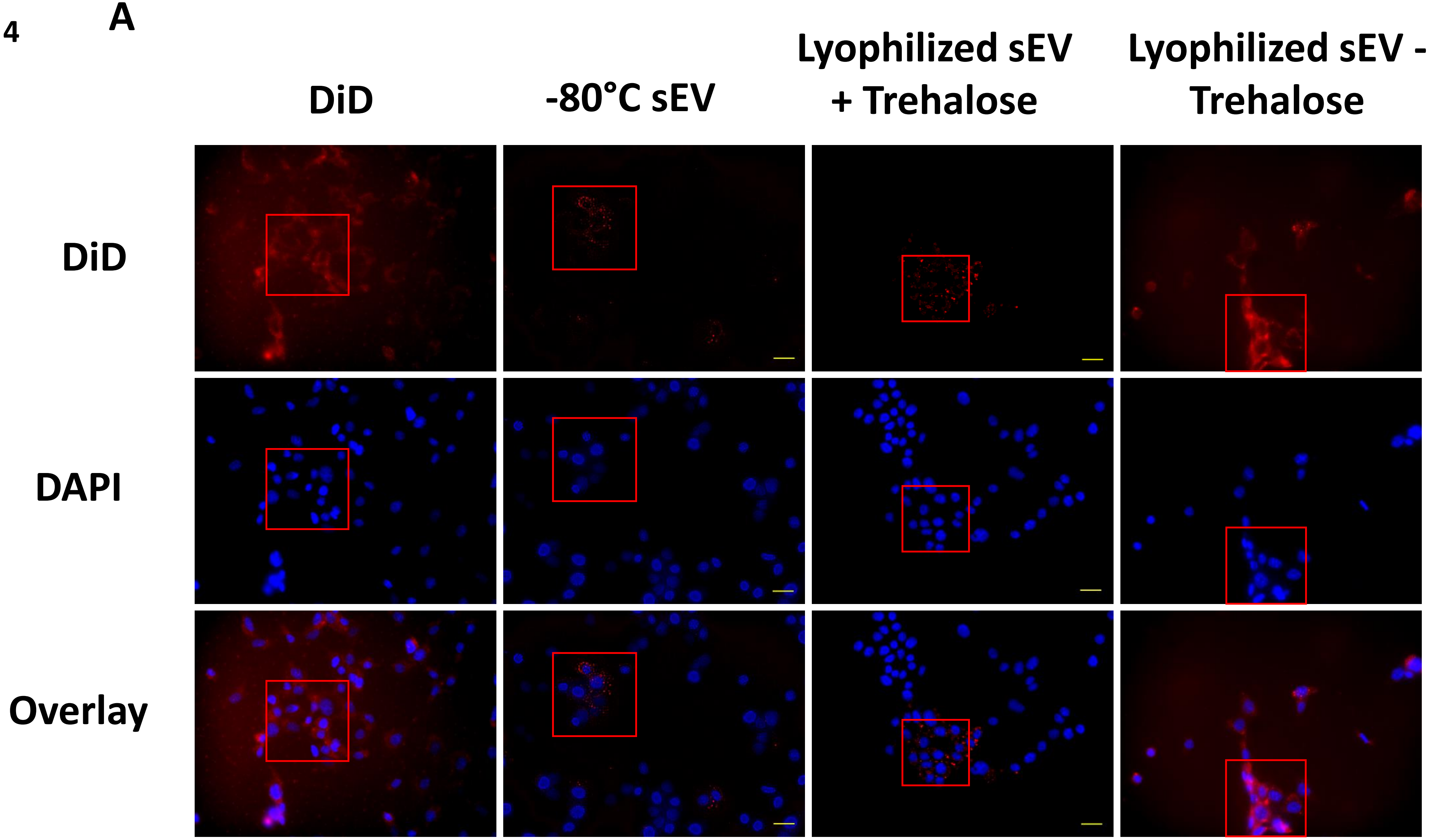

For each column, the image for DiD, DAPI and the overlay are all images of the same section of cells. The DiD is being used to stain the sEVs in this study while the DAPI stains the nucleus of the cells. The overlay shows both the DiD and DAPI in one image together to help visualize how well the cells are uptaking each type of EV. For the very first column the DiD is being used to stain the cell membrane to help demonstrate that the DiD shown in the images with cells treated with sEVs is a direct effect of the sEVs, not excess DiD. Images were cropped to provide a closer look at the sEV uptake. The red boxes indicate the cropped image that can be found in the manuscript.

Figure 4

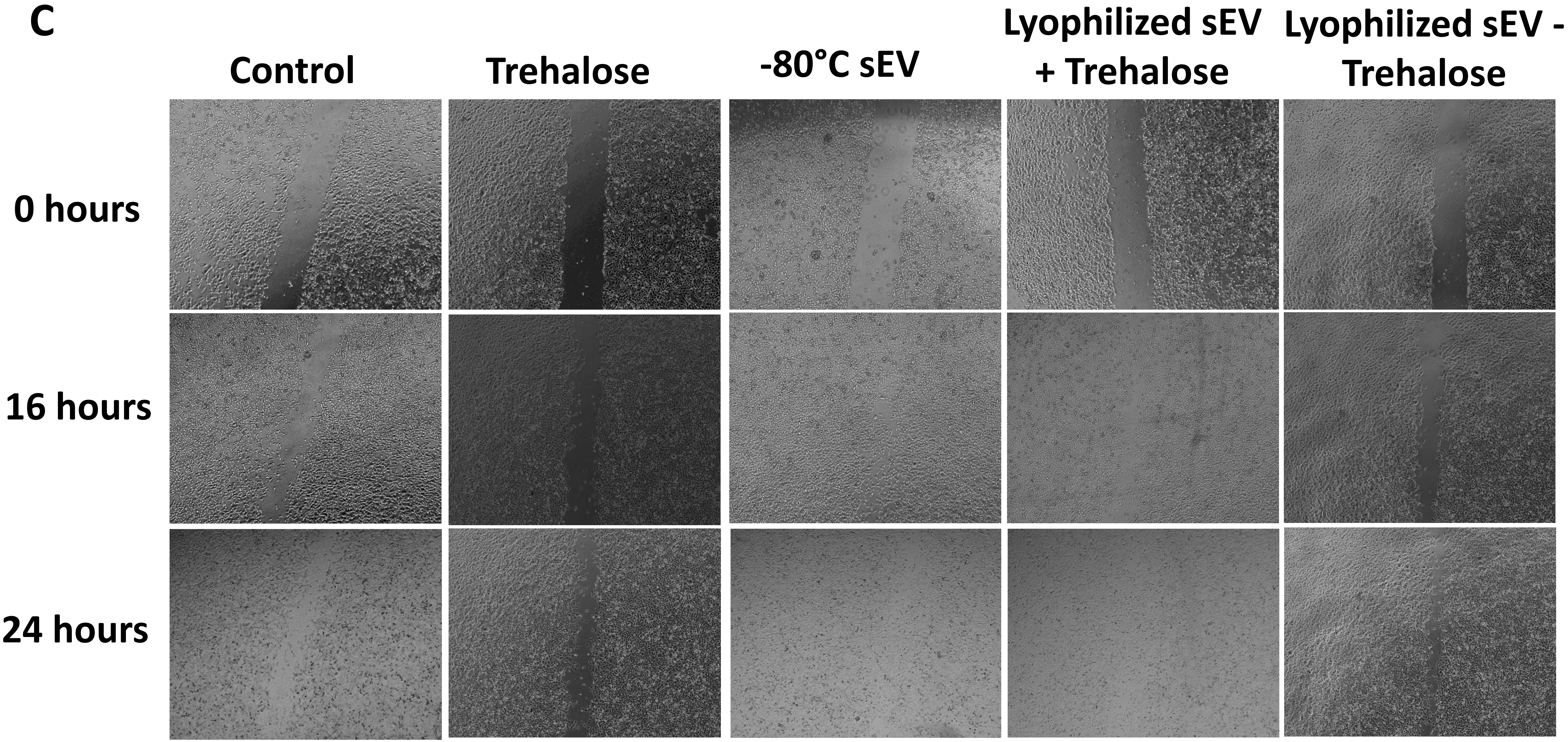

The images in each column shown in the figure above demonstrate a scratch in HT22 cells at different time intervals. The scratch shown at each time interval is the same scratch in the same section of cells to demonstrate how each treatment affects the ability of the cells to regenerate.

Figure 5

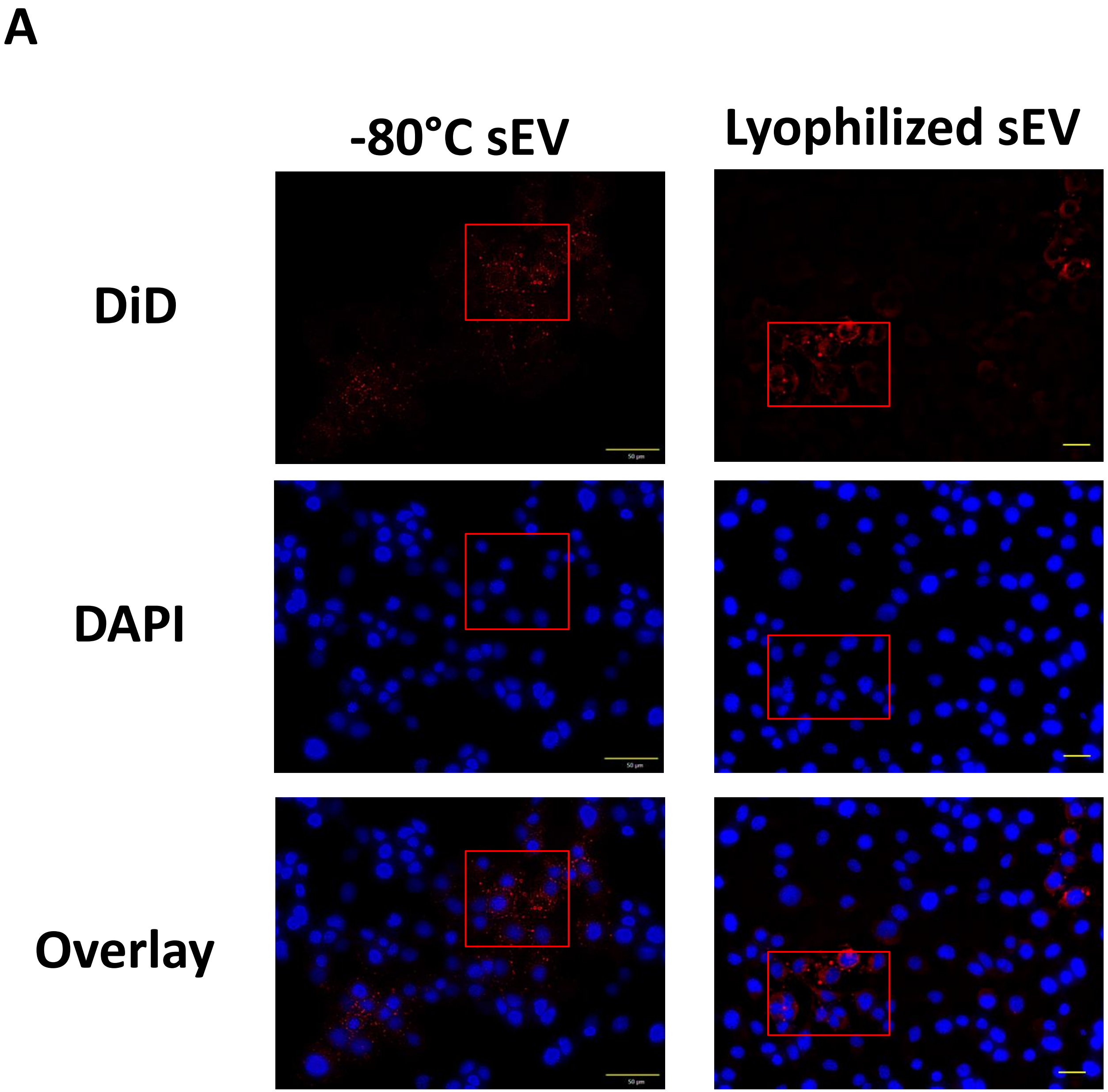

For each column, the image for DiD, DAPI and the overlay are all images of the same section of cells. The DiD is being used to stain the sEVs in this study while the DAPI stains the nucleus of the cells. The overlay shows both the DiD and DAPI in one image together to help visualize how well the cells are uptaking each type of EV. Images were cropped to provide a closer look at the sEV uptake. The red boxes indicate the cropped image that can be found in the manuscript.

Figure 5

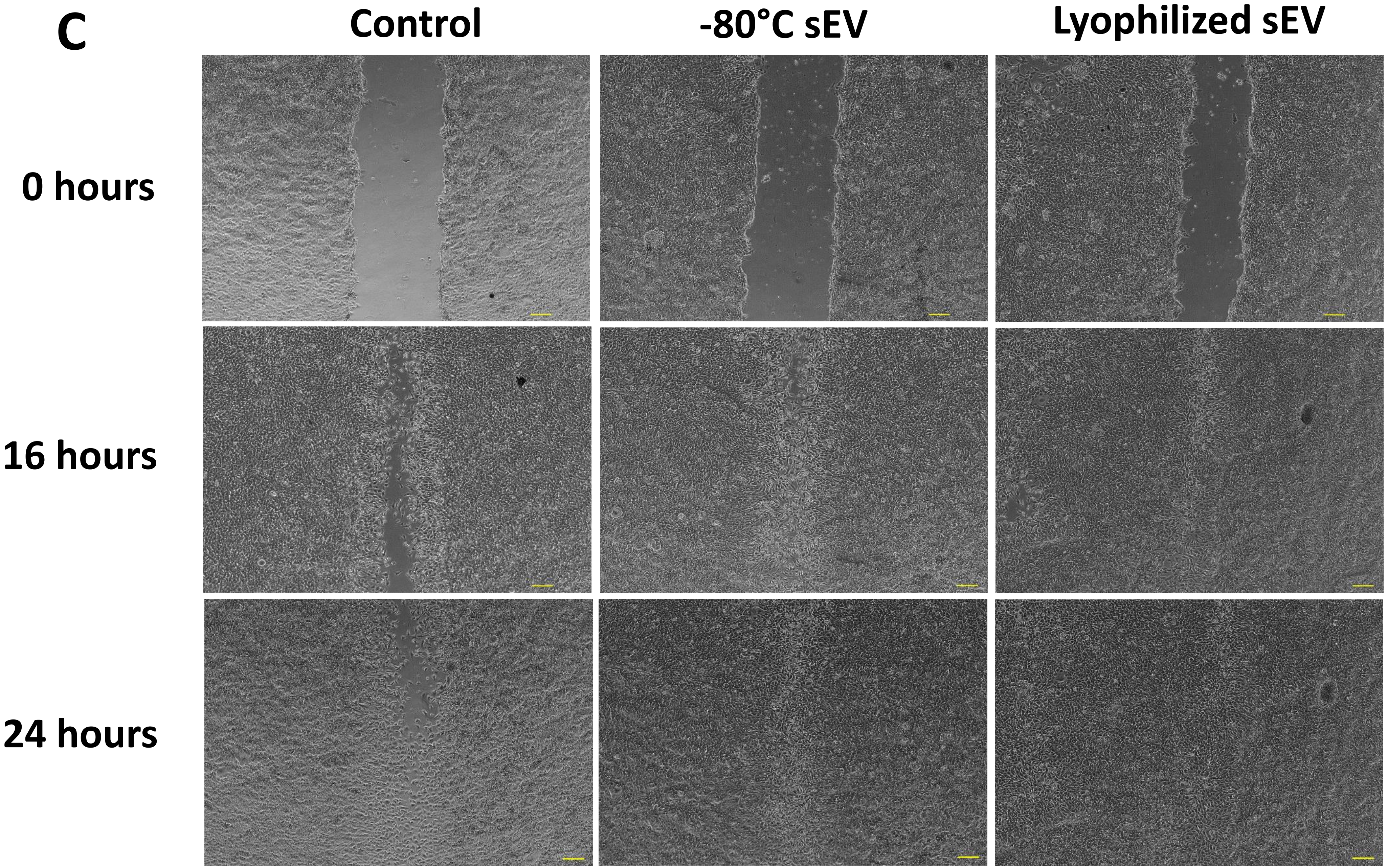

The images in each column shown in the figure above demonstrate a scratch in HT22 cells at different time intervals. The scratch shown at each time interval is the same scratch in the same section of cells to demonstrate how each treatment affects the ability of the cells to regenerate.

Figure 6

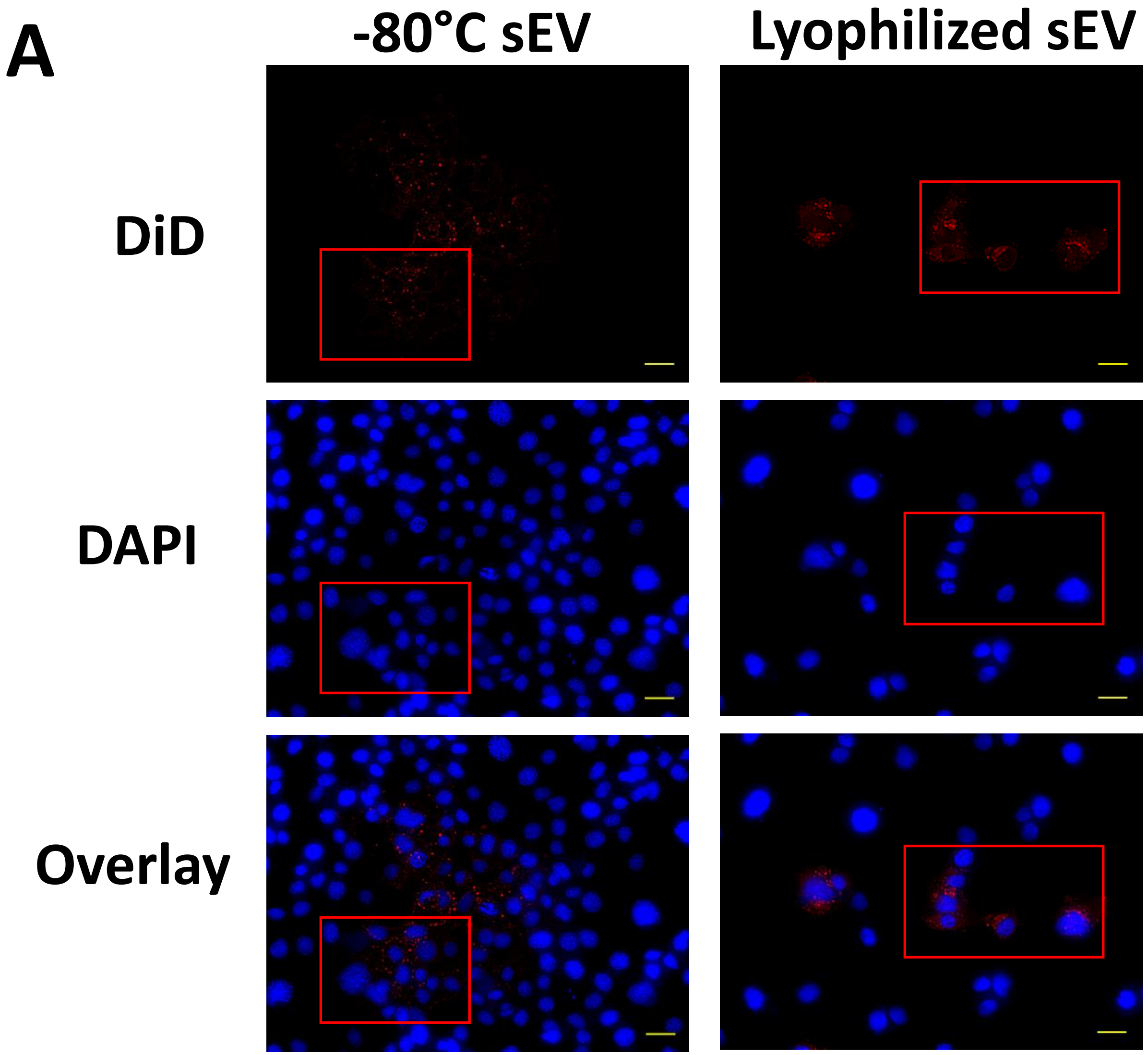

For each column, the image for DiD, DAPI and the overlay are all images of the same section of cells. The DiD is being used to stain the sEVs in this study while the DAPI stains the nucleus of the cells. The overlay shows both the DiD and DAPI in one image together to help visualize how well the cells are uptaking each type of EV. Images were cropped to provide a closer look at the sEV uptake. The red boxes indicate the cropped image that can be found in the manuscript.

Figure 6

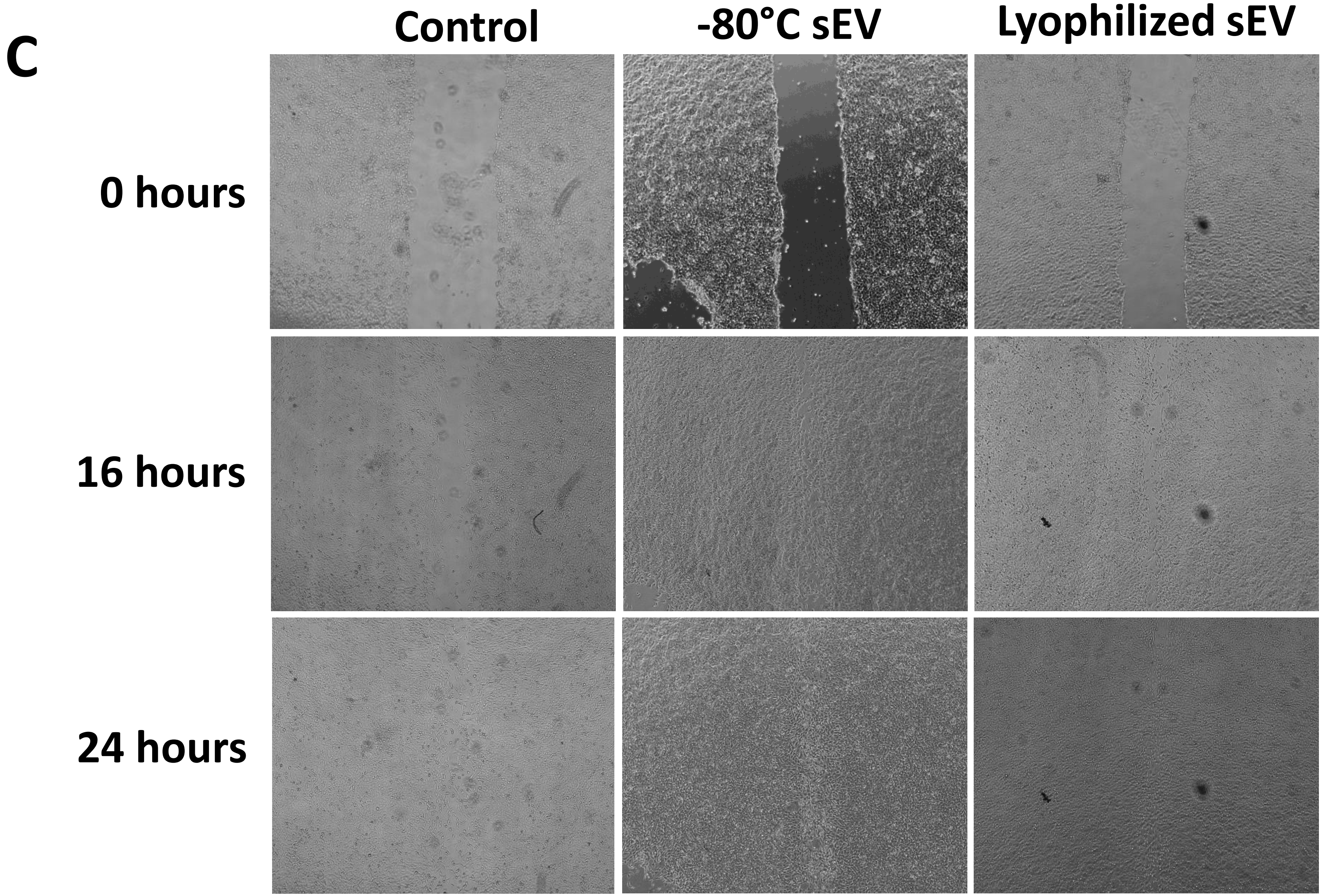

The images in each column shown in the figure above demonstrate a scratch in HT22 cells at different time intervals. The scratch shown at each time interval is the same scratch in the same section of cells to demonstrate how each treatment affects the ability of the cells to regenerate.

Figure 6

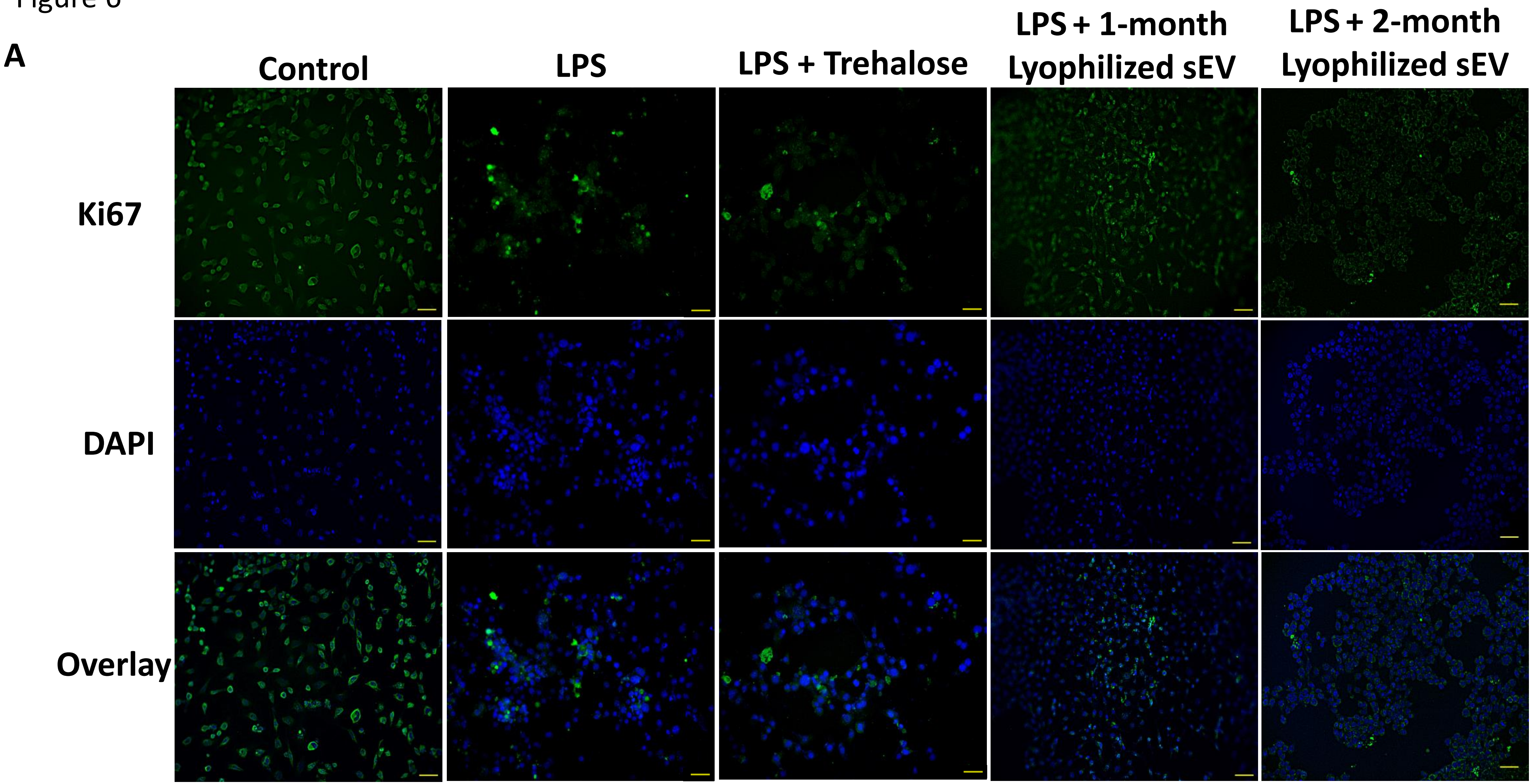

For each column, the image for Ki67, DAPI and the overlay are all images of the same section of cells. The Ki67 stains proliferating cells while the DAPI stains the nucleus of the cells. The overlay shows both the Ki67 and DAPI in one image together to help visualize how much the cell proliferation occurring from each treatment.

Figure 8

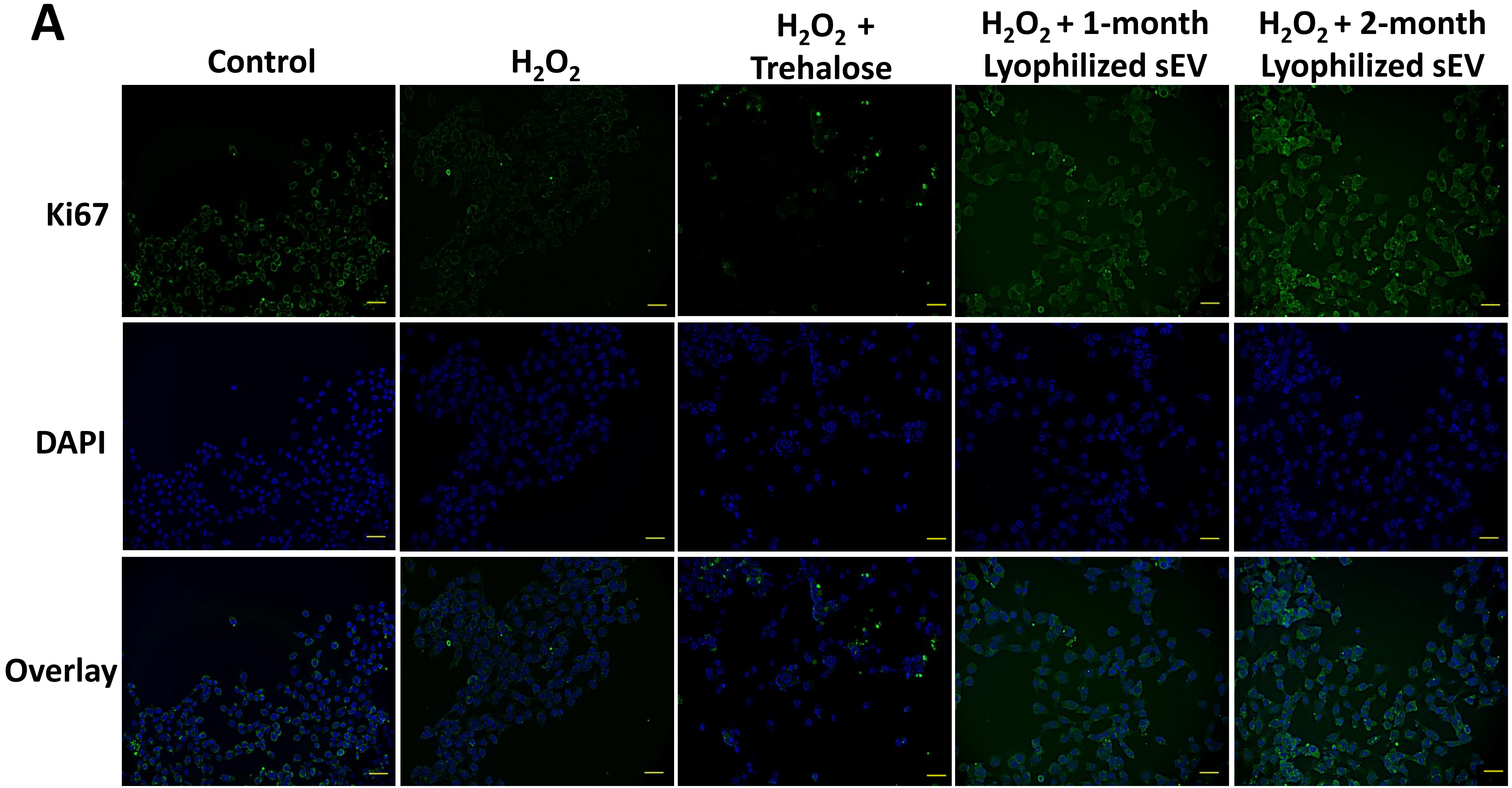

For each column, the image for Ki67, DAPI and the overlay are all images of the same section of cells. The Ki67 stains proliferating cells while the DAPI stains the nucleus of the cells. The overlay shows both the Ki67 and DAPI in one image together to help visualize how much the cell proliferation occurring from each treatment.
